# Multi-omics analysis identifies mitochondrial pathways associated with anxiety-related behavior

**DOI:** 10.1371/journal.pgen.1008358

**Published:** 2019-09-26

**Authors:** Zuzanna Misiewicz, Stella Iurato, Natalia Kulesskaya, Laura Salminen, Luis Rodrigues, Giuseppina Maccarrone, Jade Martins, Darina Czamara, Mikaela A. Laine, Ewa Sokolowska, Kalevi Trontti, Christiane Rewerts, Bozidar Novak, Naama Volk, Dong Ik Park, Eija Jokitalo, Lars Paulin, Petri Auvinen, Vootele Voikar, Alon Chen, Angelika Erhardt, Christoph W. Turck, Iiris Hovatta

**Affiliations:** 1 Molecular and Integrative Biosciences Research Program, University of Helsinki, Helsinki, Finland; 2 Department of Translational Research in Psychiatry, Max Planck Institute of Psychiatry, Munich, Germany; 3 Department of Psychology and Logopedics, Medicum, University of Helsinki, Helsinki, Finland; 4 Department of Neurobiology, Weizmann Institute of Science, Rehovot, Israel; 5 Electron Microscopy Unit, Institute of Biotechnology, University of Helsinki, Helsinki, Finland; 6 Institute of Biotechnology, University of Helsinki, Helsinki, Finland; 7 Neuroscience Center, Helsinki Institute of Life Science, University of Helsinki, Helsinki, Finland; 8 Department of Stress Neurobiology and Neurogenetics, Max Planck Institute of Psychiatry, Munich, Germany; University of California Los Angeles, UNITED STATES

## Abstract

Stressful life events are major environmental risk factors for anxiety disorders, although not all individuals exposed to stress develop clinical anxiety. The molecular mechanisms underlying the influence of environmental effects on anxiety are largely unknown. To identify biological pathways mediating stress-related anxiety and resilience to it, we used the chronic social defeat stress (CSDS) paradigm in male mice of two inbred strains, C57BL/6NCrl (B6) and DBA/2NCrl (D2), that differ in their susceptibility to stress. Using a multi-omics approach, we identified differential mRNA, miRNA and protein expression changes in the bed nucleus of the stria terminalis (BNST) and blood cells after chronic stress. Integrative gene set enrichment analysis revealed enrichment of mitochondrial-related genes in the BNST and blood of stressed mice. To translate these results to human anxiety, we investigated blood gene expression changes associated with exposure-induced panic attacks. Remarkably, we found reduced expression of mitochondrial-related genes in D2 stress-susceptible mice and in exposure-induced panic attacks in humans, but increased expression of these genes in B6 stress-susceptible mice. Moreover, stress-susceptible vs. stress-resilient B6 mice displayed more mitochondrial cross-sections in the post-synaptic compartment after CSDS. Our findings demonstrate mitochondrial-related alterations in gene expression as an evolutionarily conserved response in stress-related behaviors and validate the use of cross-species approaches in investigating the biological mechanisms underlying anxiety disorders.

## Author summary

Genetic and environmental factors contribute to the etiology of psychiatric diseases but the underlying mechanisms are poorly understood. Chronic psychosocial stress is a well-known risk factor for anxiety disorders. To identify biological pathways involved in psychosocial stress-induced anxiety and resilience to it, we used a well-characterized mouse model of chronic social defeat stress (CSDS) in two inbred mouse strains, C57BL/6NCrl (B6) and DBA/2NCrl (D2), which differ in their susceptibility to stress. We focused on the bed nucleus of the stria terminalis, a key brain region behind stress-response and anxiety, and carried out genome-wide analysis of mRNA, and miRNA expression, and protein abundance. Bioinformatic integration of these data supported differences in mitochondrial pathways as a major stress response. To translate these findings to human anxiety, we investigated blood cell gene expression in mice and in panic disorder patients exposed to fearful situations and experiencing panic attacks. Concurring with our brain findings, expression of mitochondrial pathways was also affected in mouse and human blood cells, suggesting that the observed stress response mechanisms are evolutionarily conserved. Therefore, chronic stress may critically affect cellular energy metabolism, a finding that may offer new targets for therapeutic interventions of stress-related diseases.

## Introduction

Chronic stress is a significant risk factor for human anxiety disorders [1], yet not all individuals exposed to stress develop a clinically relevant anxiety symptomatology. The underlying reasons for these differences are not yet fully understood but involve an interaction of complex genetic and environmental factors that vary among individuals resulting in stress susceptibility or resilience. The chronic social defeat stress (CSDS) model is a well-established mouse paradigm of psychosocial stress, with construct, face, discriminative, and predictive validity for stress-related disorders [2–4]. It involves 10 days of brief daily confrontation of two conspecific male mice, a resident-aggressor and an intruder who reacts with defensive, flight, or submissive behavior [5, 6]. Although all defeated mice experience stressful stimuli, only some develop stress-related symptoms, measured as social avoidance, making it an excellent model to investigate mechanisms associated with susceptibility and resilience. We have previously shown that genetic factors strongly affect the behavioral outcome of the CSDS, since different inbred mouse strains vary in the proportion of susceptible and resilient animals as well as in their stress coping behaviors [4].

Anxiety disorders are common stress-associated psychiatric disorders [7, 8]. They are characterized by an excessive physiological and emotional response in the absence of real threat or imminent danger. Among the most debilitating anxiety disorders is panic disorder, which involves sudden recurrent surges of intense fear and discomfort called panic attacks [9]. However, panic attacks are not exclusive to panic disorder, but also frequent in other anxiety disorders. They are typically associated with severe perceived physical and mental stress, feeling of loss of control and avoidance behavior. Epidemiological studies show a major impact of both cumulative and specific life events or stressors, such as threat or psychosocial interpersonal life events, on the development of all anxiety disorders [10, 11], including panic disorder [12].

The development of much-needed novel targets for therapeutic intervention of anxiety disorders is limited by the ignorance of the molecular and cellular mechanisms associated with events that initiate and maintain pathological anxiety. The phenotypic heterogeneity of human populations and the high variability of environmental influences [13], along with a limited access to brain tissue samples, make it difficult to identify the biological basis of anxiety disorders. These challenges can, to some extent, be controlled in animal models. Cross-species approaches are therefore expected to reveal specific biological mechanisms underlying anxiety disorders [14]. They can be especially powerful for multi-omics studies allowing hypothesis-free identification of the most significant biological pathways associated with specific exposures, while at the same time probing the effects of different genetic backgrounds on the outcomes. Therefore, the integration of multiple levels of information [15] and the translation of the results from animal models to the human condition are critical for the success of cross-species approaches.

Omics-based approaches require the investigation of etiologically relevant tissue. The CSDS model has been used to study transcriptomic effects of chronic psychosocial stress in brain regions classically associated with anxiety and depression-related behaviors, including the medial prefrontal cortex, hippocampus, amygdala, and nucleus accumbens [4, 16]. In addition, converging lines of anatomical, physiological, and mechanistic evidence suggest that the bed nucleus of the stria terminalis (BNST) functions as the center of integration for limbic information [17], monitoring the genesis of long-term responses to anxiety [18], such as anticipatory anxiety. The BNST is an especially relevant brain region for stress-related anxiety. It has been suggested that it plays a role in valence surveillance by processing salient information on physical and social contexts, collected through its numerous projections throughout the brain [17]. However, only few anxiety studies have generated large-scale genomics data from the BNST [19].

To identify the core differentially expressed molecules and pathways underlying pathological anxiety and resilience to it, we employed a multi-omics approach in mice. We applied the CSDS model to induce anxiety-related phenotypes and to identify molecular markers for susceptibility and resilience in male mice. We used two inbred strains, the largely stress-resilient C57BL/6NCrl (B6) and stress-susceptible DBA/2NCrl (D2), due to strong genetic background effects in the mouse stress response [4, 20]. We investigated gene (RNA-seq), microRNA (miRNA-seq), and protein (LC-MS/MS) expression differences in the BNST between the stress-resilient, stress-susceptible, and control mice. As a translational effort, we also carried out RNA-seq from mouse post-CSDS blood, and longitudinal microarray-based gene expression profiling from blood cells of panic disorder patients who experienced high degrees of stress and anxiety due to exposure to phobic situations. To identify converging anxiety-related gene sets and pathways, we conducted pathway and gene set enrichment analyses of the data sets from mouse BNST samples, and mouse and human blood cells. Altogether, our results indicate anxiety induces a genetically-controlled evolutionarily conserved response in mitochondrial pathways.

## Results

### Genetic background has a strong effect on the behavioral response to CSDS

**Fig 1:**
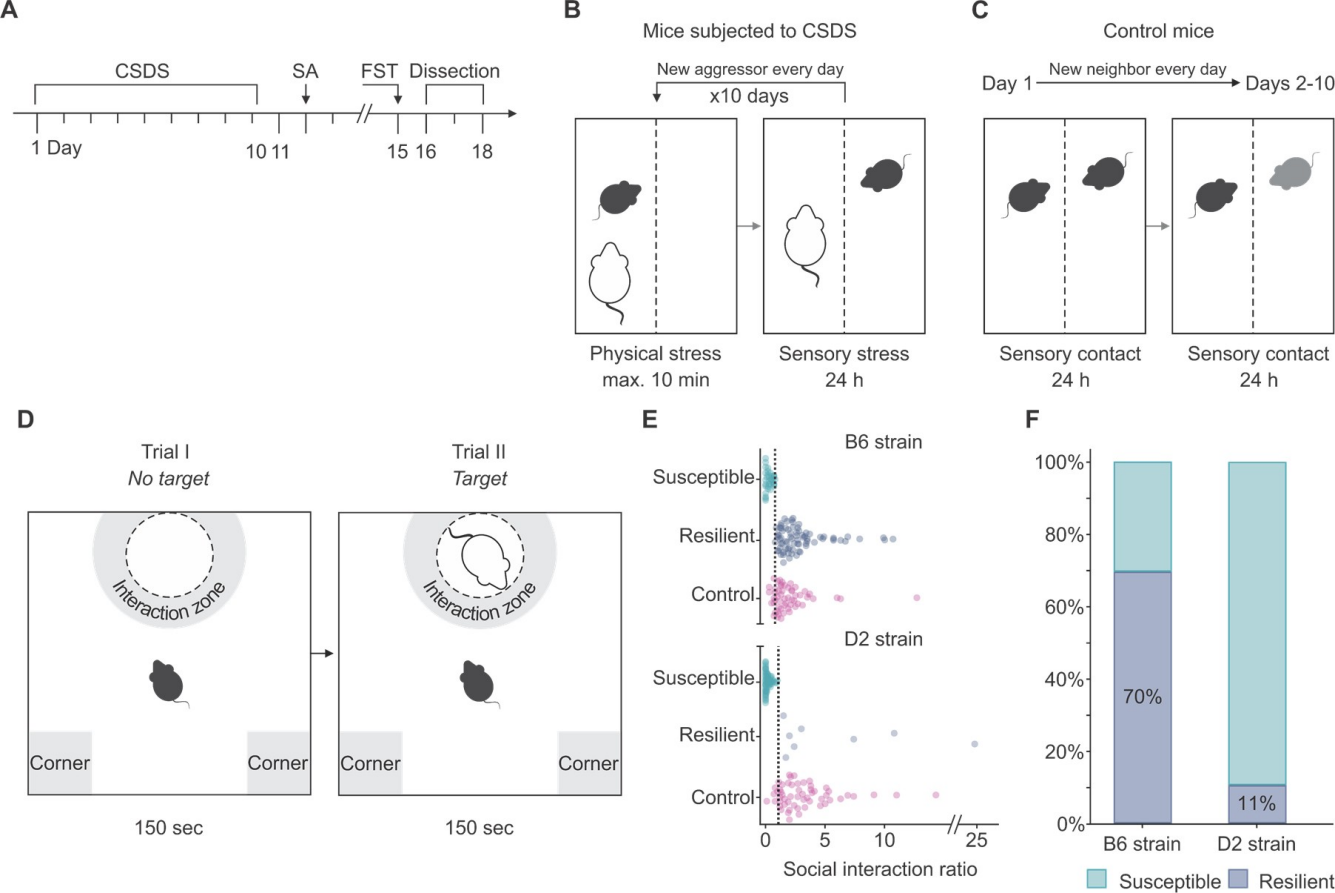
Genetic background has a strong effect on the behavioral response to chronic social defeat stress (CSDS). (A) Timeline of behavioral experiments including CSDS, social avoidance (SA) test, forced swim test (FST), and tissue collection. (B) Schematic illustration of 10-day CSDS. The protocol involved a daily confrontation of max. 10 min of two conspecific mice, the experimental mouse in the defeated group (black) and the aggressor mouse (white), followed by sensory contact through a clear perforated plexiglass divider for the remaining of the 24 hours. As indicated by the black arrow, the defeated mice met a new aggressor every day during the 10 days of CSDS. (C) Schematic illustration of the control condition. Two control mice were placed in a single cage separated by a clear perforated plexiglass divider. As indicated by the black arrow, every 24 hours the control mouse was placed into a cage with a new unfamiliar neighbor mouse. Unlike the defeated mice, the control mice were never in physical contact with their neighbors. (D) Schematic illustration of trial I and II of the SA test. (E) SA test results showing social interaction ratios. *n* = B6: Susceptible = 34, Resilient = 78, Control = 56; D2: Susceptible = 62, Resilient = 8, Control = 56. Outliers criterion: IQR > 3. (F) Percentage of mice resilient and susceptible to stress. *n* = B6: Susceptible = 34, Resilient = 78 D2: Susceptible = 62, Resilient = 8. B6: C57BL/6NCrl; D2: DBA/2NCrl; FST: Forced swim test; IQR: interquartile range; SA: social avoidance test.

To study how genetic background affects the behavioral response to chronic psychosocial stress, we subjected B6 and D2 mice to a 10-day CSDS, followed by the social avoidance (SA) test 24 hours later (Fig 1, S1 Table). Since these strains differ in their innate social avoidance levels [4], we evaluated the behavior of the defeated mice in comparison to the same-strain controls. We divided the defeated group of animals and defined stress-susceptible mice as those with social interaction (SI) ratios below one standard deviation from the mean SI ratio of the same-strain controls, as previously described [4]. We classified all other defeated mice with ratios above those values, i.e. resembling controls, as resilient (Fig 1E). The strains showed distinct response to stress since 89% of D2 mice, but only 30% of B6 mice, presented social avoidance behavior, being susceptible to chronic psychosocial stress (Pearson’s chi-square, χ^2^ = 60.38, *P* = 7.76E^-14^) (Fig 1F).

CSDS also significantly affected locomotor behavior of mice. Both B6 and D2 susceptible mice moved significantly less than same-strain controls (*P* = 0.003 and *P* = 0.002, respectively) during the no-target trial, i.e., the trial without the social target mouse (Fig 1D), of the SA test. D2 resilient mice moved significantly more than D2 susceptible mice (*P* = 0.003), while no such difference was observed in the B6 strain (*P* = 0.443) (S1 Table).

To assess if chronic psychosocial stress affects the duration to cease escape-oriented behavior in the face of an acute stressor, we performed the forced swim test (FST) five days after the end of CSDS. The latency to immobility during the FST, used as a measure of active stress coping [21, 22], was highly correlated with the SI ratio in the D2 defeated mice (*r* = 0.920, *P* = 0.009) (S1 Table). In other words, defeated D2 mice with higher resilience to psychosocial stress presented a more active coping strategy than mice with higher social avoidance. We did not observe similar correlations in the D2 control group (*r* = -0.130, *P* = 0.759) or in the B6 control or defeated mice (*r* = -0.079, *P* = 0.691 and *r* = 0.026, *P* = 0.852, respectively) (S1 Table). We also found that CSDS had a significant effect on the body weight of the D2, but not the B6 mice (S1 Fig). Overall, these results suggest that genetic background has a strong effect on stress susceptibility, with the D2 strain being more susceptible to stress-induced social avoidance than the B6 strain. Furthermore, the two strains used different strategies to cope with stress, as demonstrated by their differences in locomotor and escape-oriented behavior after, and body weight development throughout, the CSDS.

### Divergent BNST differential gene expression and protein abundance in B6 and D2 strains following CSDS

**Fig 2:**
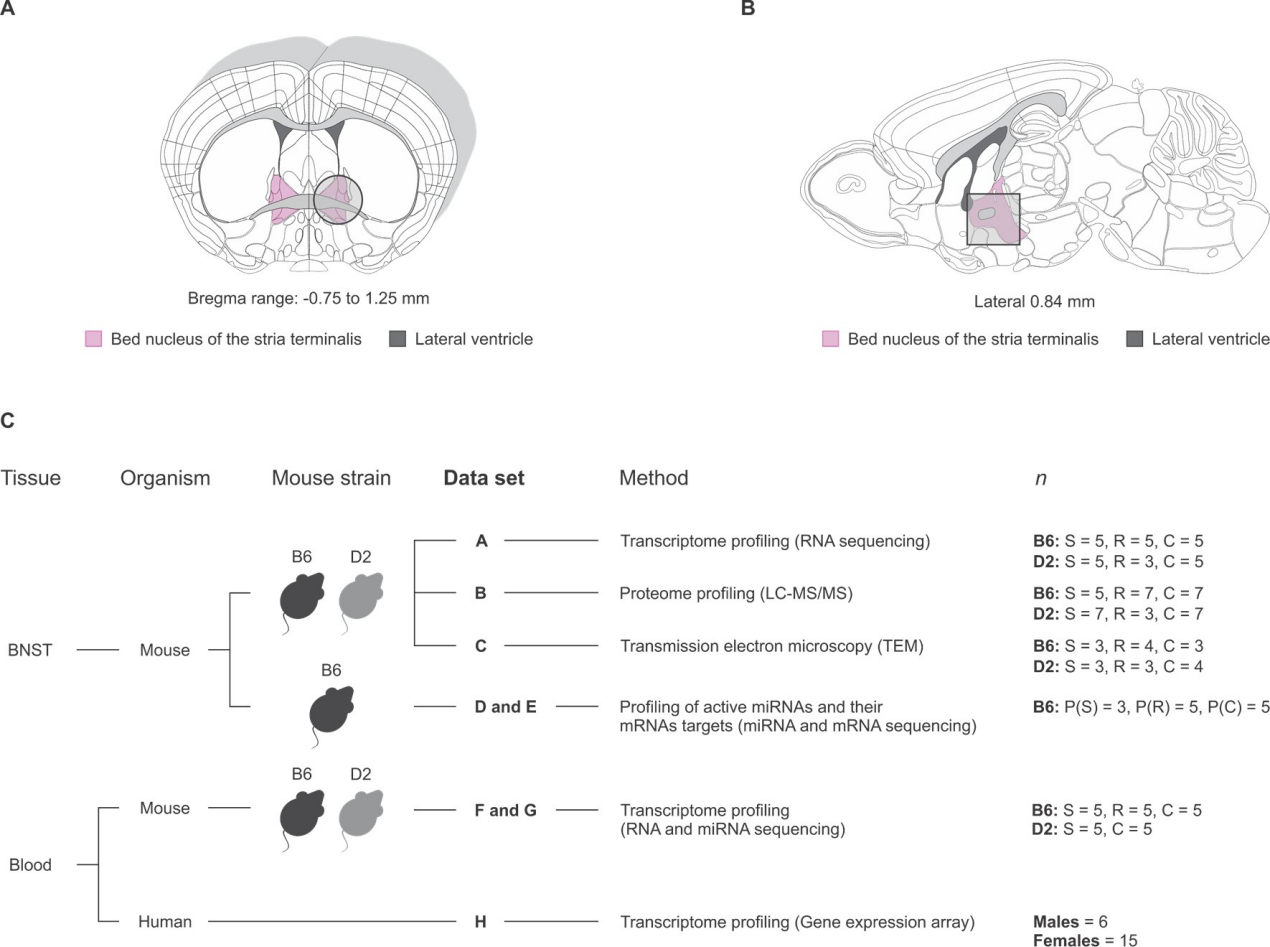
Overview of the BNST tissue collection and analyzed data sets. (A-B) BNST was dissected from both hemispheres by punching the indicated area (shaded area outlined with a circle or a square) for (A) transcriptomic, proteomic, profiling of active miRNA and their target mRNAs, and (B) transmission electron microscopy (TEM) analyses. Figure outlines are based on Allen Mouse Brain Atlas [103, 104]. The bregma for outline shown in figure (A) is 0.38. (C) Schematic of analyzed data sets. B6: C57BL/6NCrl; C: control; D2: DBA/2NCrl; LC-MS/MS: liquid chromatography–tandem mass spectrometry; R: resilient; S: susceptible.

To identify stress-associated transcriptomic and proteomic signatures in the B6 and D2 mouse strains, we profiled the BNST, a key brain region regulating anxiety. Profiling was conducted approximately one week following completion of the CSDS, when we sacrificed all mice and dissected BNSTs for analyses (Fig 2A and 2B). We then carried out both gene expression (RNA-seq) and proteomic (liquid chromatography-tandem mass spectrometry) profiling to identify differentially expressed mRNAs (data set A) and proteins (data set B) (Fig 2C). Additionally, in the B6 strain, we performed Argonaute 2 RNA immunoprecipitation-sequencing (AGO2 RIP-seq) of active microRNAs (miRNAs) and their mRNA targets (data sets D and E, respectively). AGO2 is the catalytic component of the RNA-induced silencing complex (RISC). Only mature miRNAs can be incorporated into RISC in the presence of AGO2, and serve as a guide molecule for silencing their target mRNAs [23]. Thus, AGO2 RIP-seq identifies only those miRNAs and their target mRNAs that are bound to the RISC at the time of tissue collection, providing additional specificity compared to the sequencing of all cellular miRNAs and mRNAs. Data sets A and B were collected from the same cohorts of animals, with each cohort being equally divided by the SI ratios between transcriptomic and proteomic experiments. AGO2 RIP-seq (data set D and E) was performed from an additional cohort. In all data sets, we compared the stress-resilient, stress-susceptible, and same-strain control mice. For the resilient and susceptible groups, we selected mice representing the phenotypic extremes, i.e., those with the highest and lowest SI ratios, respectively. Unless stated otherwise, “differentially expressed” (DE) mRNAs, miRNAs and proteins were defined as *P* < 0.05 and |FC| ≥ 1.2. For all individual findings of DE mRNAs, miRNAs and proteins, we report both nominal *P*-values and *P*-values adjusted for multiple testing by the modified Benjamini–Hochberg method (*Padj)* as defined in [24]. The total numbers of genes and proteins DE at *P* < 0.05 and *Padj* < 0.05 levels for each data set are presented in S2 Table.

#### Common DE genes show opposite expression patterns in stress-susceptible B6 and D2 mice

**Fig 3:**
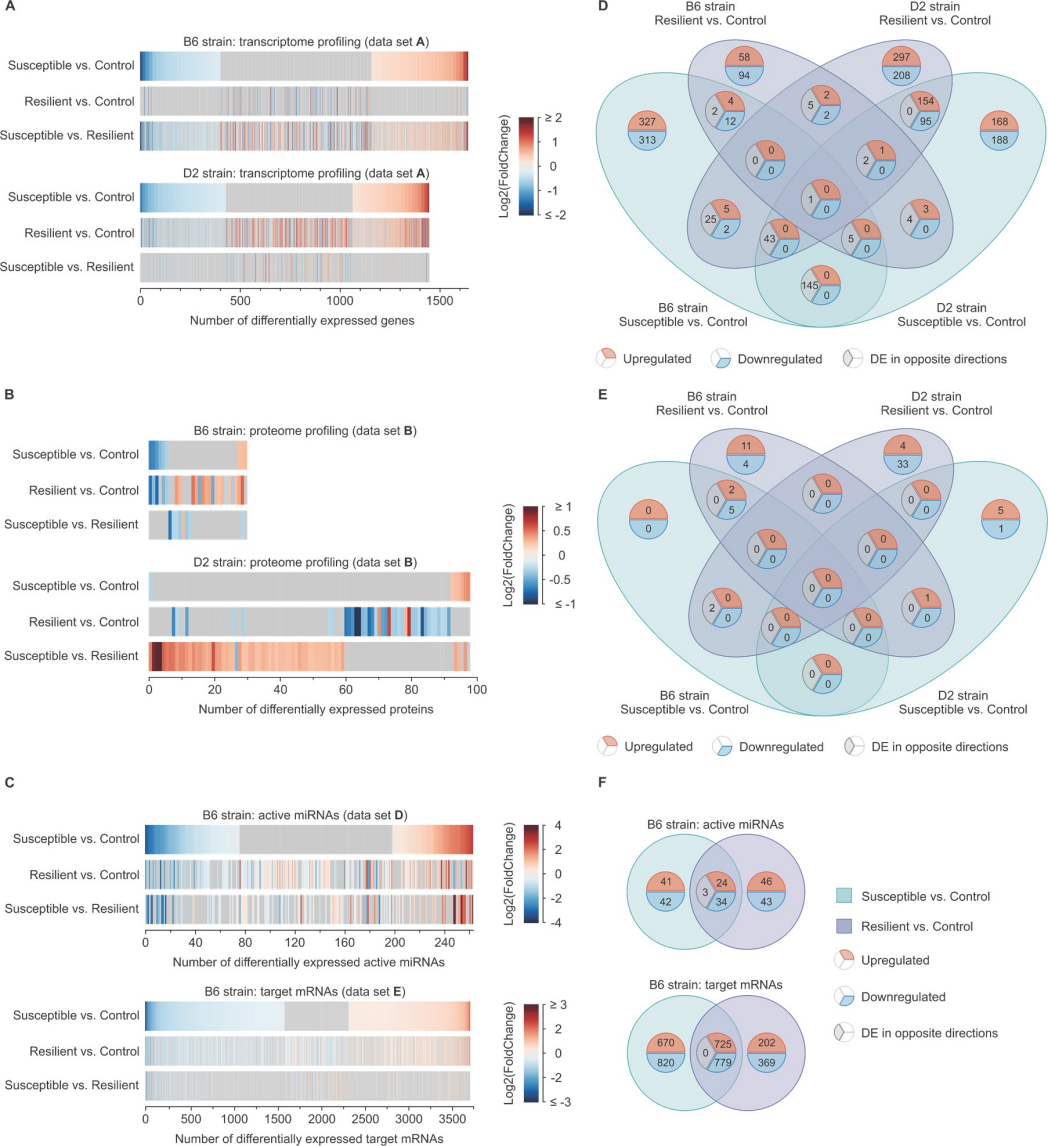
Distinct transcriptional stress response in the BNST of the D2 and B6 strains following CSDS. (A-C) Union heatmaps showing fold changes (FCs) of (A) significantly differentially expressed (DE; *P* < 0.05 and |FC| ≥ 1.2) genes (data set A), (B) proteins (data set B), and (C) active miRNAs and their target mRNAs (data sets D and E) following CSDS. The genes are rank-ordered by the FCs in the susceptible vs. control comparison, separately for each strain and–omics data set. (D-F) Venn diagrams showing overlap between significantly DE (D) genes (data set A), (E) proteins (data set B), and (F) active miRNAs and their target mRNAs (data sets D and E) following CSDS. B6: C57BL/6NCrl; CSDS: chronic social defeat stress; D2: DBA/2NCrl.

We first examined the transcriptional stress response in the BNST of stress-susceptible and resilient B6 and D2 mice. The overall number of unique DE genes in both strains was similar (*n*: B6 = 1638, D2 = 1441; Fig 3A and 3D, S2 Fig), of which only 194 were common between the strains when stress-susceptible mice were compared to the controls. Notably, all of them were DE in opposite directions between B6 and D2 mice (Fig 3A and 3D). Since none of the individual genes were significantly DE after multiple testing correction, we performed Gene set enrichment and Gene Ontology (GO) term enrichment analyses to investigate whether these genes are part of common biological pathways. We found that these nominally DE genes were significantly enriched for mitochondrial and translation-related gene sets (S3 Table) and GO terms (S4 Table) at *P*_*FDR*_ < 0.05. Thus, our results reveal highly divergent gene expression patterns in the two strains after CSDS.

#### Discrete changes in protein abundance after CSDS between the strains

We next examined protein abundance differences of B6 and D2 mice in the BNST after CSDS. In total, we detected 117 DE proteins in at least one of the comparisons in either or both strains (Fig 3B and 3E). A single protein, phosphatase 1 regulatory subunit 1B (PPP1R1B), also known as dopamine- and cAMP-regulated neuronal phosphoprotein (DARPP-32), was nominally DE in the susceptible compared to the resilient mice in both strains (*P* = 0.011 and *Padj* = 0.652 in B6 strain; *P* = 4.95E^-9^ and *Padj =* 1.34E^-6^ in the D2 strain). The protein was less abundant in the stress-susceptible B6 mice and more abundant in the D2 mice compared to the stress-resilient groups. Therefore, also the protein expression patterns after chronic psychosocial stress were significantly different between the B6 and D2 strains.

#### Capturing the active miRNA-mRNA interactions in the BNST

To identify stress-responsive miRNAs, we carried out parallel RNA-sequencing of active AGO2-associated miRNAs and their bound mRNA targets [25] in the B6 strain. We detected 233 DE miRNAs and 3565 DE mRNA targets (Fig 3C and 3F, S2 Table) between the studied groups (susceptible, resilient and control). Although we observed largely discrete patterns of miRNA expression in stress-susceptible and resilient B6 mice, a significant number of miRNAs were DE in the same direction in both groups indicating a general stress-related response. This finding might reflect the previously described double-role of miRNAs in both restoring homeostasis, upon stress-related environmental changes, and enforcing new phenotype-specific gene expression patterns following CSDS to better adjust to the stressful events [26].

### Integrative pathway and gene set enrichment analyses reveal differential expression of mitochondria-related genes and proteins in the mouse BNST

#### DE genes and proteins are enriched for pathways related to mitochondrial function

After collecting the individual mRNA, miRNA and protein expression data sets, we carried out several integrative analyses to identify biological pathways significantly affected by chronic psychosocial stress. First, we conducted Ingenuity Pathway Analysis (IPA) and gene set enrichment analysis (GSEA) for DE genes (data set A) and proteins (data set B) of the susceptible vs. control, resilient vs. control and susceptible vs. resilient comparisons in B6 and D2 strains. On the transcriptome level, the most significantly dysregulated pathway was the mitochondria-related oxidative phosphorylation pathway. It was upregulated in susceptible B6 mice compared to controls (Z-score = 4.90, *P*_*FDR*_ = 3.38E^-10^), but downregulated in the same comparison in the D2 strain (Z-score = -5.57, *P*_*FDR*_ = 5.47E^-18^), a result which was replicated by the GSEA (S3 Fig). Opposite molecular signatures for the susceptible vs. control comparison were also observed for the second most significantly dysregulated pathway, the eIF2 signaling pathway responsible for translational control, with upregulation in the B6 (Z-score = 4.47, *P*_*FDR*_ = 2.67E^-8^) and downregulation in D2 strain (Z-score = -3.61, *P*_*FDR*_ = 1.78E^-3^). On the protein level, the converging cAMP-mediated signaling and dopamine-DARPP32 feedback in cAMP signaling pathways were predicted to be dysregulated (*P*_*FDR*_ < 0.01) in both B6 and D2 strains. The dopamine-DARPP32 feedback in cAMP signaling pathway was upregulated in the B6 resilient mice in comparison to the same-strain controls (*P*_*FDR*_ = 0.001) and the D2 susceptible vs. resilient mice (*P*_*FDR*_ = 0.003).

**Fig 4:**
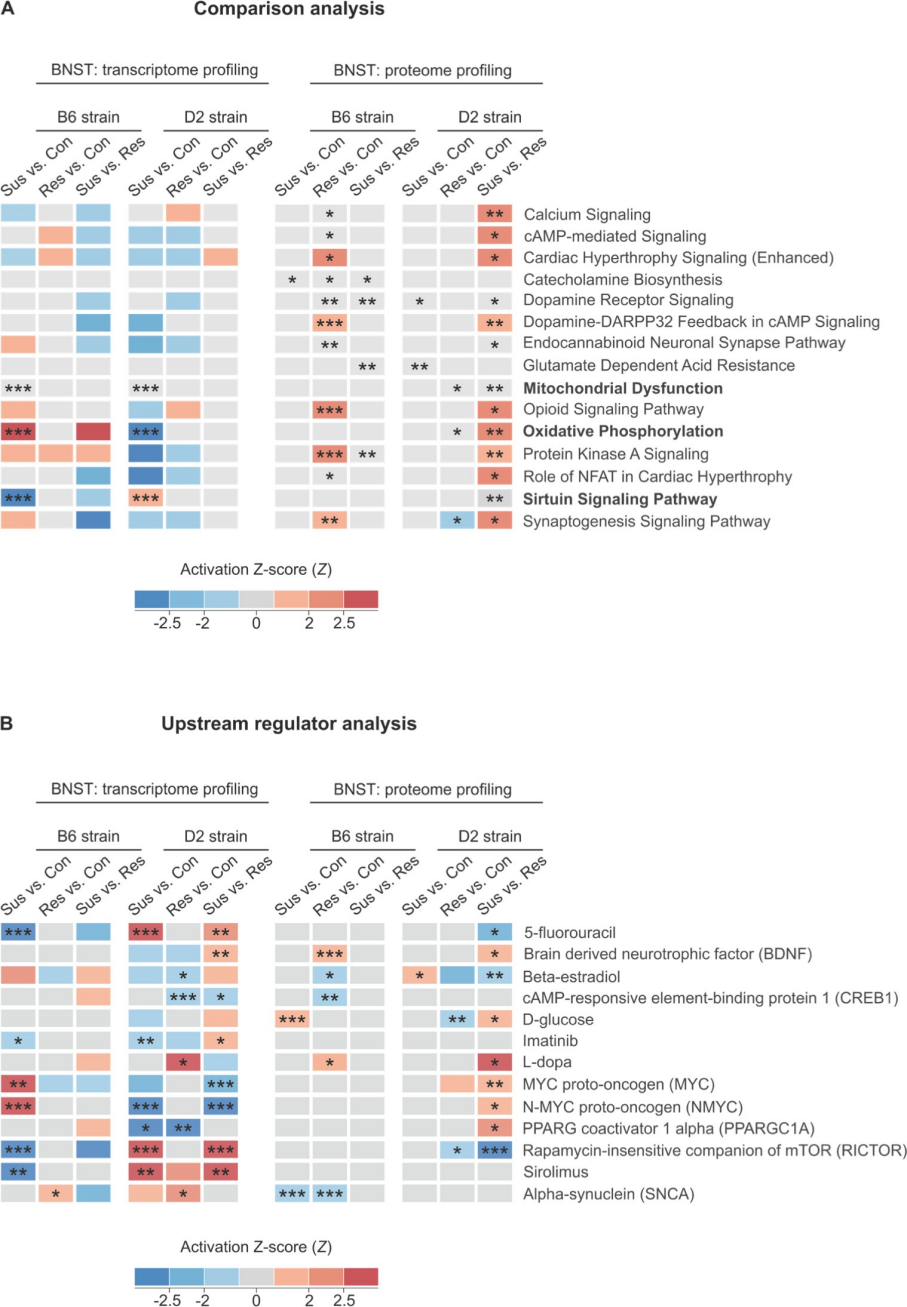
Transcriptomic and proteomic analyses implicate dysregulation of mitochondria-related canonical pathways in the BNST of B6 and D2 mice following CSDS. Merged heatmap showing (A) significantly dysregulated canonical pathways and (B) upstream regulators from the Ingenuity Pathway Analysis (IPA) database ordered alphabetically. Mitochondria-related canonical pathways (written in bold) were found to be significantly dysregulated (*P*_*FDR*_ < 0.05) on both gene and protein levels. The IPA Z-score, predicting the activation (red) or inhibition (blue) of the respective signaling pathway or upstream regulator cluster, is marked in color. B6: C57BL/6NCrl; cAMP: cyclic adenosine monophosphate; Con: control; D2: DBA/2NCrl; DARPP-32: dopamine- and cAMP-regulated phosphoprotein; NAFT: nuclear factor of activated T-cells; PPARG: peroxisome proliferator-activated receptor gamma; Res: resilient; Sus: susceptible. *P_*FDR*_ < 0.05, ** *P*_*FDR*_ < 0.01, *** *P*_*FDR*_ < 0.001.

To identify the most widely stress-affected pathways, we carried out an integrative analysis of the 12 transcriptomic and proteomic comparisons (Fig 4A). We found 15 pathways with at least two of the 12 comparisons significantly DE (*P*_*FDR*_ < 0.05) and at least four of the 12 comparisons nominally significantly DE (*P* < 0.05). Notably, three mitochondria-related pathways (Fig 4A, written in bold) were significantly dysregulated (*P*_*FDR*_ < 0.05) on both transcriptome and proteome levels. Taken together, these results show significant enrichment of pathways related to mitochondrial function and transcriptional control after CSDS, and suggest that their directionality, i.e., upregulation in the B6 susceptible mice and downregulation in the D2 susceptible mice in comparison to the same-strain controls, is affected by the genetic background.

#### Common putative upstream regulators of DE genes and proteins

To identify transcriptional regulators, which could explain the observed changes in gene and protein expression after CSDS, we performed the IPA Upstream Regulator analysis (Fig 4B). It examines the data set for known targets of transcription regulators and compares the directionality of the gene expression differences to the information included in the Ingenuity Knowledge Base [27]. On the transcriptome level, rapamycin-insensitive companion of mTOR (RICTOR) was a potential upstream regulator with the highest bioinformatic activation score in both B6 and D2 strains among all findings in the analysis (*P*_*FDR*_ < 0.001). RICTOR was predicted to be inhibited in the B6 susceptible mice in comparison to controls (Z-score = -4.84, *P*_*FDR*_ = 6.37E^-12^) and activated in the same comparison in the D2 strain (Z-score = 6.55, *P*_*FDR*_ = 8.96E^-14^). RICTOR’s function in cell growth has been extensively described [29]. The protein is also known to be involved in dendritic branch formation [28, 29]. Furthermore, neuronal knockout mice of *Rictor* have reduced pre-pulse inhibition, a schizophrenia-like symptom [30].

A reverse pattern of activation was predicted for the N-myc proto-oncogene protein (N-MYC) cluster. On the transcriptome level, N-MYC was predicted to be activated in B6 susceptible mice in comparison to controls (Z-score = 3.09, *P*_*FDR*_ = 8.57E^-4^) but inhibited in the same comparison in the D2 strain (Z-score = -2.51, *P*_*FDR*_ = 2.13E^-4^). N-MYC regulates cell proliferation and growth, apoptosis, and mitochondrial biogenesis [31].

The target genes within the beta-estradiol cluster were predicted to be significantly downregulated (*P*_*FDR*_ = 0.015) in the D2 resilient mice in comparison to the controls on the transcriptome level. On the proteome level, the same genes within the beta-estradiol cluster were significantly downregulated in the B6 resilient mice in comparison to the B6 controls (*P*_*FDR*_ = 0.015) and significantly inhibited in the D2 susceptible mice in comparison to the resilient group (Z-score = -2.40, *P*_*FDR*_ = 0.008). Thus, as previously shown [32], some stress-associated DE genes may be under the regulation of hormones.

#### Lower levels of miR-34c and miR-99b/100, and higher levels of miR-15b in B6 mice after CSDS

To identify active DE miRNAs and their mRNA targets in the BNST of B6 mice following CSDS, we carried out AGO-RIP-seq (data sets D and E; Fig 2C) and employed the IPA microRNA Target Filter tool [27]. It uses experimentally validated interactions from TarBase [33], miRecords [34] and micro-RNA related findings from the published literature [27], and microRNA-mRNA interactions predicted by TargetScan [35]. We observed 72 (resilient vs. control), 59 (susceptible vs. controls), and 36 (susceptible vs. resilient) DE miRNAs, which were either experimentally validated or computationally predicted (high confidence) to repress from one to multiple co-immunoprecipitated AGO2-bound DE mRNAs (S5 Table). Only matching pairs of miRNAs and mRNAs that were DE in the same direction were included in the analysis, since they may form functional AGO2-bound miRNA-mRNA pairs. Next, to further increase the confidence of our findings, we selected miRNA-mRNA interactions identified by more than two IPA microRNA Target Filter sources in at least one comparison. We found nominally lower levels of miR-34c and higher levels of miR-15b in both resilient and susceptible mice in comparison to the controls (Table 1). Additionally, we observed nominally lower levels of miR-99b/100 in the stress-susceptible mice compared to both control (FC = -1.50, *P* = 6.69E^-5^, *Padj* = 0.024) and resilient (FC = -1.22, *P* = 0.011, *Padj* = 0.183) mice, as well as in the resilient mice in comparison to the controls (FC = -1.22, *P* = 0.005, *Padj* = 0.076) (S5 Table and Table 1). The DE genes *Epdr1*, *Ntrk2*, *Cldn11*, and *Ppp3ca* were predicted targets of miR-99b/100 (S5 Table). Of these genes, *Cldn11* was also nominally differentially expressed in the BNST susceptible vs. control transcriptomic analysis in both strains (data set A; FC = 1.61, *P* = 0.015, *Padj* = 0.630 and FC = -1.68, *P* = 0.009, *Padj* = 0.608), confirming it as a robust stress-affected gene.

**Table 1:** Differentially expressed miRNAs and their predicted differentially expressed target genes in the BNST following CSDS.

Comparison	miRNA	Fold change	*P* (*Padj*)	Tool name	Gene
B6 Resilient - Control	miR-34c	-1.77	0.041 (0.187)	Ingenuity Expert Findings, TargetScan, miRecords	*Jag1*
	miR-126a	1.21	0.011 (0.098)	Ingenuity Expert Findings, TargetScan, miRecords	*Irs1*
	miR-15b	1.33	0.002 (0.043)	TarBase, TargetScan, miRecords	*Dmtf1*
B6 Susceptible - Control	miR-34c	-2.84	0.004 (0.085)	Ingenuity Expert Findings, TargetScan, miRecords	*Jag1*
	miR-33	1.25	0.047 (0.240)	Ingenuity Expert Findings, TargetScan, miRecords	*Abca1*
	miR-18a	3.22	0.026 (0.195)	Ingenuity Expert Findings, TargetScan, miRecords	*Esr1*
	miR-15b	1.35	0.003 (0.077)	TarBase, TargetScan, miRecords	*Dmtf1*
B6 Susceptible -Resilient	miR-99b/100	-1.22	0.011 (0.183)	Ingenuity Expert Findings, TargetScan, miRecords	*Igf1r*

*Abca1*: ATP-binding cassette transporter; B6: C57BL/6NCrl; *Dmtf1*: Cyclin D binding Myb-like transcription factor 1; *Esr1*: Estrogen receptor 1; *Igf1r*: Insulin-like growth factor 1 receptor; *Jag1*: Jagged1; *Irs1*: Insulin receptor substrate 1.

#### Integrative multi-omics analysis implicates dysregulation of PPP1R1B and CYCS after CSDS

**Fig 5:**
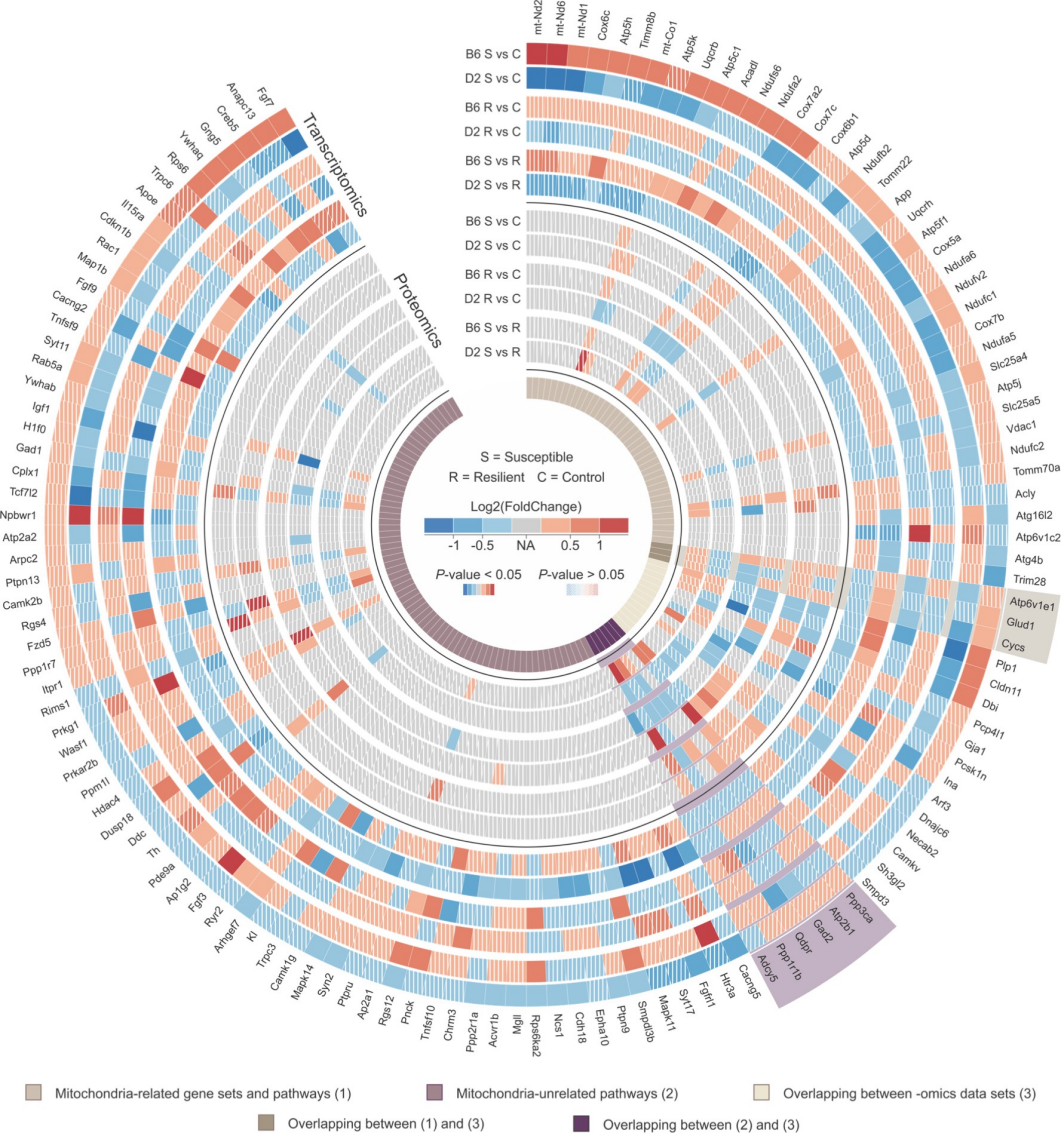
Common differentially expressed (DE) genes and proteins included in the enriched mitochondria-related gene sets and pathways in the BNST of B6 and D2 mice following CSDS. Merged heatmap showing the expression fold change (FC) and *P*-value of resilient vs. control, susceptible vs. control and susceptible vs. resilient comparisons from the BNST transcriptomic and proteomic analyses (data sets A and B, respectively). Only DE molecules (*P* < 0.01) in at least one of the 12 comparisons are shown. “Mitochondria-related gene sets and pathways” include “Mitochondrial Dysfunction,” “Oxidative Phosphorylation” and “Sirtuin Signaling Pathway” shown in Fig 4A and all gene sets shown in S3 Fig. All remaining canonical pathways included in Fig 4A are referred to as “Mitochondria-unrelated pathways”. Genes and proteins significantly DE in the same comparison between at least two BNST data sets are referred to as *overlapping between–omic data sets* and were selected based on results shown in S6 Table. Molecules with *P* > 0.05, are shown in diagonal stripe pattern, while those with *P* < 0.05 are shown with solid colors. Molecules not present in a data set are shown in gray. B6: C57BL/6NCrl; C: control; D2: DBA/2NCrl; R: resilient; S: susceptible.

**Fig 6:**
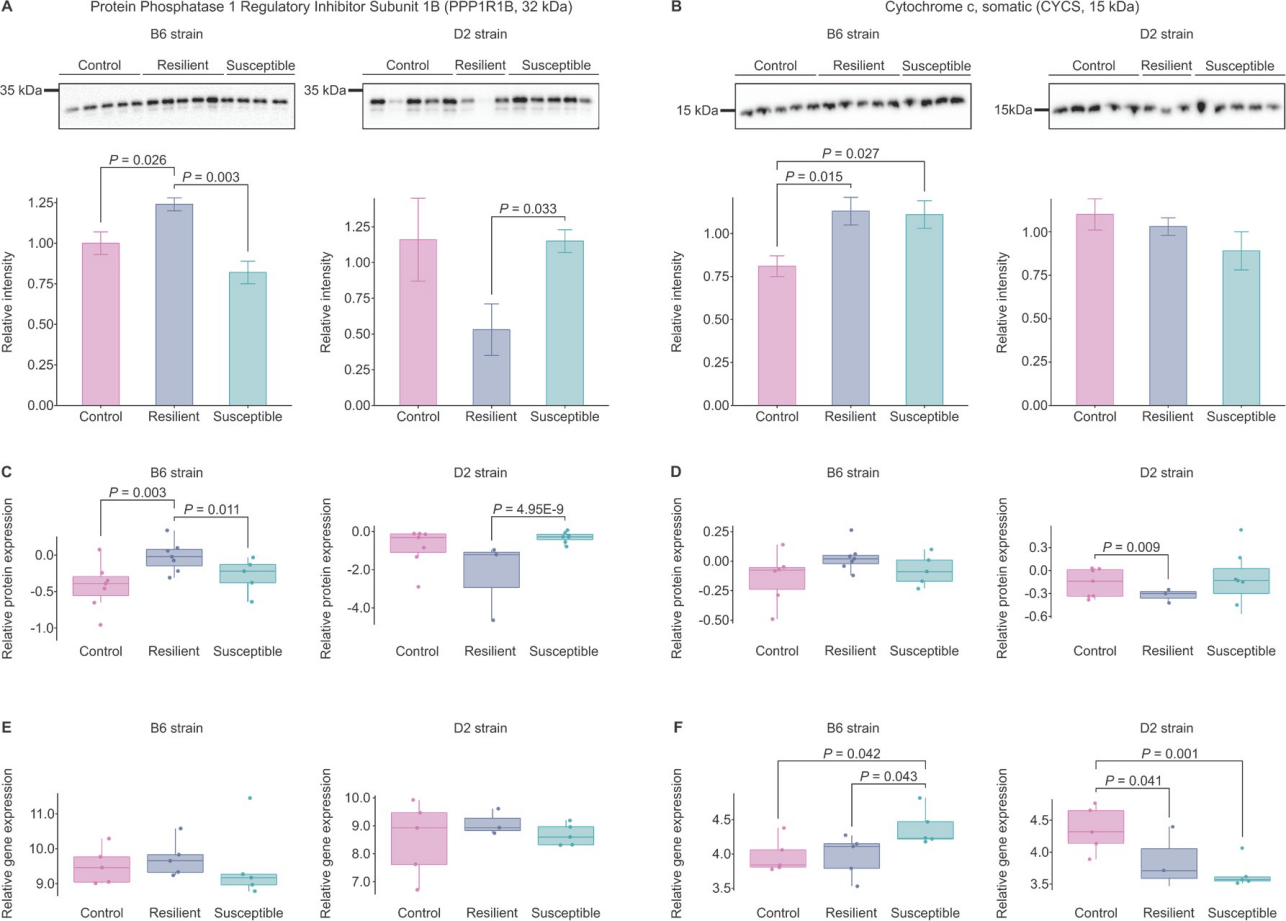
Protein phosphatase 1 Regulatory Subunit 1B (PPP1R1B) and cytochrome c somatic (CYCS) show opposite patterns of expression in the BNST of B6 and D2 strains following CSDS. (A-B) Western blot (WB), (C-D) relative protein (data set B; LC-MS/MS), and (E-F) gene expression (data set A; RNA sequencing) analysis of total PPP1R1B and CYCS in mouse BNST following exposure to CSDS. Mean values ± S.E.M. B6: C57BL/6NCrl; D2: DBA/2NCrl; LC-MS/MS: liquid chromatography–tandem mass spectrometry.

To identify the key stress-related molecules shared between transcriptomic and proteomic data sets, we focused on the most significantly dysregulated transcriptomic or proteomic pathways from the IPA analysis (*P*_*FDR*_ < 0.05; Fig 4A) and GSEA (*P*_*FDR*_ < 0.05; S3 Fig) (Fig 5). We asked if any molecule specific to those pathways and gene sets shows similar pattern of expression in at least one of the comparisons (susceptible vs. control, resilient vs. control, or susceptible vs. resilient) between at least two BNST data sets (Fig 2C, S6 Table). We found three common molecules, *Cycs*, *Glud1*, and *Atp6v1e1*, in mitochondria-related gene sets and pathways, and six molecules, *Adcy5*, *Ppp1r1b*, *Qdpr*, *Gad2*, *Atp2b1* and *Ppp3ca*, in other canonical mitochondria-unrelated pathways (Fig 5). We selected two of these proteins, PPP1R1B and CYCS (cytochrome c somatic), which have been previously associated with psychiatric disorders, including anxiety disorders [36–39], for validation by Western blot analysis (Fig 6).

PPP1R1B acts as an integrator of dopaminergic and glutamatergic signaling, and elevated levels of its truncated isoform have been observed in schizophrenia, bipolar disorders, major depression, and poor cognitive functioning [40]. Western blot analysis confirmed the proteomic results of significantly more abundant PPP1R1B levels in B6 resilient mice in comparison to the control (*P* = 0.026) and susceptible (*P* = 0.003) groups, while in the D2 strain, it was significantly less abundant in resilient compared to susceptible mice (*P* = 0.033) (Fig 6A). Furthermore, PPP1R1B protein levels detected by Western blot analysis correlated significantly with SI ratios in the B6 strain (*r* = 0.689, *P* = 0.006), but not in the D2 strain (*r* = -0.244, *P* = 0.422) (S4 Fig).

CYCS is a central element of the electron transport chain in mitochondria [41, 42]. Western blot analysis showed that CYCS was more abundant in both the B6 susceptible (*P* = 0.027) and resilient mice (*P* = 0.015) in comparison to controls (Fig 6B). This was in contrast to the transcriptomic data (data set A), where *Cycs* was expressed at nominally higher levels in susceptible compared to both the control (*P* = 0.042, *Padj* = 0.686) and resilient mice (*P* = 0.043, *Padj* = 0.593) (Fig 6F). We did not observe differences in the CYCS abundance in the D2 strain by Western blot, although its abundance was lower in the resilient compared to control mice in the LC-MS/MS data (data set B) (Fig 6D). Overall, the Western blot analysis confirmed the divergent expression of both PPP1R1B and CYCS observed in the transcriptomic and proteomic analyses following CSDS.

### Mitochondrial pathways identified by gene expression profiling of blood cells from stressed mice and panic disorder patients with exposure-induced panic attacks

We next asked if the same pathways and gene sets that we found to be stress-responsive in the mouse BNST, could be identified from an accessible tissue, i.e. blood cells, and whether these pathways and gene sets were also DE in blood of humans with anxiety. One week after CSDS, we collected blood samples from stress-susceptible, resilient, and control B6 and D2 mice, and carried out RNA-seq and miRNA-seq (data sets F and G, respectively). Blood samples were collected from the same mice that were used for BNST RNA-seq (data set A), except for D2 resilient mice where we did not obtain a sufficient amount of blood. As a translational human anxiety disorder data set, we collected samples from panic disorder patients who underwent an exposure intervention. We selected panic disorder as our translational target to concentrate on a phenotypically homogeneous sample. We collected blood samples at baseline, 1 h after anxiety peak during exposure and 24 h after exposure-induced panic attack, and carried out microarray-based gene expression profiling.

#### B6 and D2 strains show opposite blood cell gene expression patterns following CSDS

**Fig 7:**
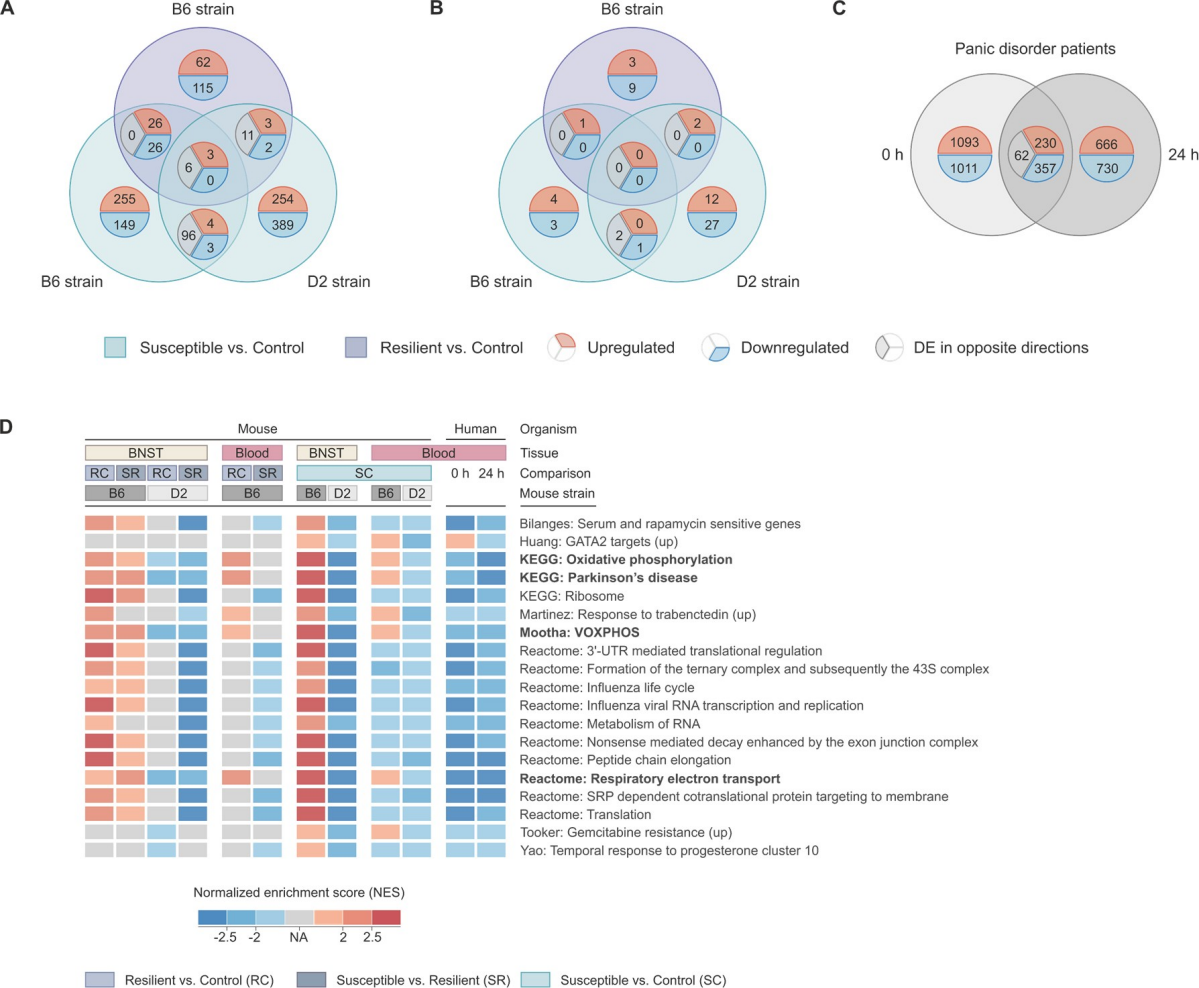
Converging analysis of blood cell gene expression data from stressed mice and panic disorder patients with exposure-induced panic attacks. (A-C) Venn diagrams showing overlap between significantly differentially expressed (A) mouse genes (data set F; *P* < 0.05 and |FC| ≥ 1.2), (B) mouse miRNAs (data set G; *P* < 0.05 and |FC| ≥ 1.2), and (C) genes (data set H; *P* < 0.05) in panic disorder (PD) patients’ blood cells. (D) Merged heatmap from gene set enrichment analysis showing the significantly enriched gene sets (*P*_*FDR*_ < 0.25) present in at least 50% of all comparisons. Gene sets which did not pass the cut-off are marked in gray (NA). A positive (or negative) NES for a given gene set implicates its overrepresentation at the top (or bottom, respectively) of the ranked list of upregulated (or downregulated, respectively) genes. Gene sets are ordered alphabetically. B6: C57BL/6NCrl; BNST: bed nucleus of the stria terminalis; D2: DBA/2NCrl.

The blood cell differential gene expression analysis in the B6 and D2 stress-susceptible mice compared to the same-strain controls identified 568 nominally DE genes in the B6 and 771 nominally DE genes in the D2 strain (S2 Table). The majority of them (91.1%, *n* = 102) were expressed in opposite directions between the strains (Fig 7A), similarly to the BNST data (data set A). To ask which biological pathways these DE genes belong to, we carried out hypergeometric statistics using C2 curated gene sets [43, 44] and GO term enrichment analysis. We found significant enrichment (*P*_*FDR*_ < 0.05) of more than 100 gene sets, with the highest number of common DE genes associated with Alzheimer’s disease gene set (*n* = 17) (S7 and S8 Tables). The average expression levels of genes that were nominally differentially expressed in the BNST and expressed in blood cells (*n* = 788), correlated significantly (Pearson’s correlation, *r* = 0.410, *P* = 2.93E^-33^), confirming a highly similar transcriptome response in both the BNST and the blood cells after CSDS.

To determine which miRNAs are differentially expressed in blood cells in response to CSDS, we performed miRNA-seq followed by DE analysis (Fig 7B). Of the 64 DE miRNAs, only three were differentially expressed in both B6 and D2 mice: miR-148a, miR-181b, and miR-592 (S9 Table). While miR-592 was expressed at a nominally lower level in both B6 and D2 stress-susceptible mice in comparison to the same-strain controls (FC = -1.71, *P* = 0.021, *Padj* = 0.797), miR-148a and miR-181b were expressed at nominally higher levels in B6 susceptible (FC = 1.34, *P* = 0.012, *Padj* = 0.731 and FC = 1.66, *P* = 0.004, *Padj* = 0.526, respectively), but lower levels in D2 susceptible mice (FC = -1.26, *P* = 0.042, *Padj* = 0.328 and FC = -1.41, *P* = 0.038, *Padj* = 0.325, respectively), compared to controls. Additionally, miR-181b was expressed at nominally higher levels in B6 susceptible compared to resilient mice (FC = 1.57, *P* = 0.008, *Padj* = 0.992). Two of the DE miRNAs, miR-34c and miR-3076, were found in the same comparison (B6 resilient vs. control) in both BNST (D) and blood (G) data sets. Notably, miR-34c was expressed at a nominally lower level in B6 resilient mice in comparison to the controls in both tissues (FC = -2.49, *P* = 0.040, *Padj* = 0.955 and FC = -1.77, *P* = 0.041, *Padj* = 0.187, in blood and the BNST, respectively). Of these three miRNAs, miR-148a has been associated with panic disorder [45], and miR-181b proposed as a non-invasive diagnostic marker for schizophrenia [46].

#### Genome-wide response in panic disorder patients’ blood data

We next examined the number of DE probes (*P* < 0.05) in panic disorder patients’ blood cells (data set H), collected directly and 24 h after exposure-induced panic attack in comparison to baseline measurement. Of all 47291 analyzed probes, 5791 (corresponding to 4149 genes) were nominally significantly DE in at least one time point. We observed a higher number of DE genes immediately after the exposure-induced panic attack (*n* = 2753) than 24 h after (*n* = 2045) (Fig 7C). A large number of the identified DE genes (*n* = 649) was common between the comparisons. Following differential expression analysis, the probes were annotated to HGNC Gene Symbols and DE genes (*P* < 0.05) and analyzed for overrepresentation of GO terms. The most significant GO term of Biological process shared between both time points was “Translation” (*P*_*FDR*_ = 1.72E^-7^ at 0 h and *P*_*FDR*_ = 1.97E^-3^ at 24 h). The fifth most significant shared term was “Oxidative phosphorylation” (*P*_*FDR*_ = 8.24E^-5^ at 0 h and *P*_*FDR*_ = 1.39E^-3^ at 24 h) (S10 Table). Overall, we found several enriched GO terms (*P*_*FDR*_ < 0.05) related to translational control and mitochondria in both time points in comparison to the baseline.

#### Changes in mitochondria-related pathways are a feature of anxiety in mice and humans

We next integrated results from the GSEA performed with BNST and blood cell transcriptomic data from stressed mice (data sets A and F) and panic disorder patients exposed to panic attacks (data set H). Due to the large number of data sets, differences in tissue source and organisms, a recommended exploratory false discovery rate (FDR) of 25% [43] was applied. Again, we found enrichment of several mitochondria and translational control-related gene sets (Fig 7D). Intriguingly, the enriched gene sets in the panic disorder patients’ blood cells, both directly and 24 h after exposure-induced panic attack in comparison to the baseline, showed a similar pattern to D2 mice exposed to CSDS, including downregulation of the genes in the enriched pathways. Thus, although we found opposite gene expression patterns in the two mouse strains, the pattern of the highly stress-susceptible mouse strain resembled that of panic disorder patients. These results suggest that the systemic transcriptional regulation of mitochondrial pathways is an evolutionarily-conserved response to anxiety in both humans and mice.

### Susceptibility to psychosocial stress is associated with differences in mitochondrial morphology and number in the B6 BNST

**Fig 8:**
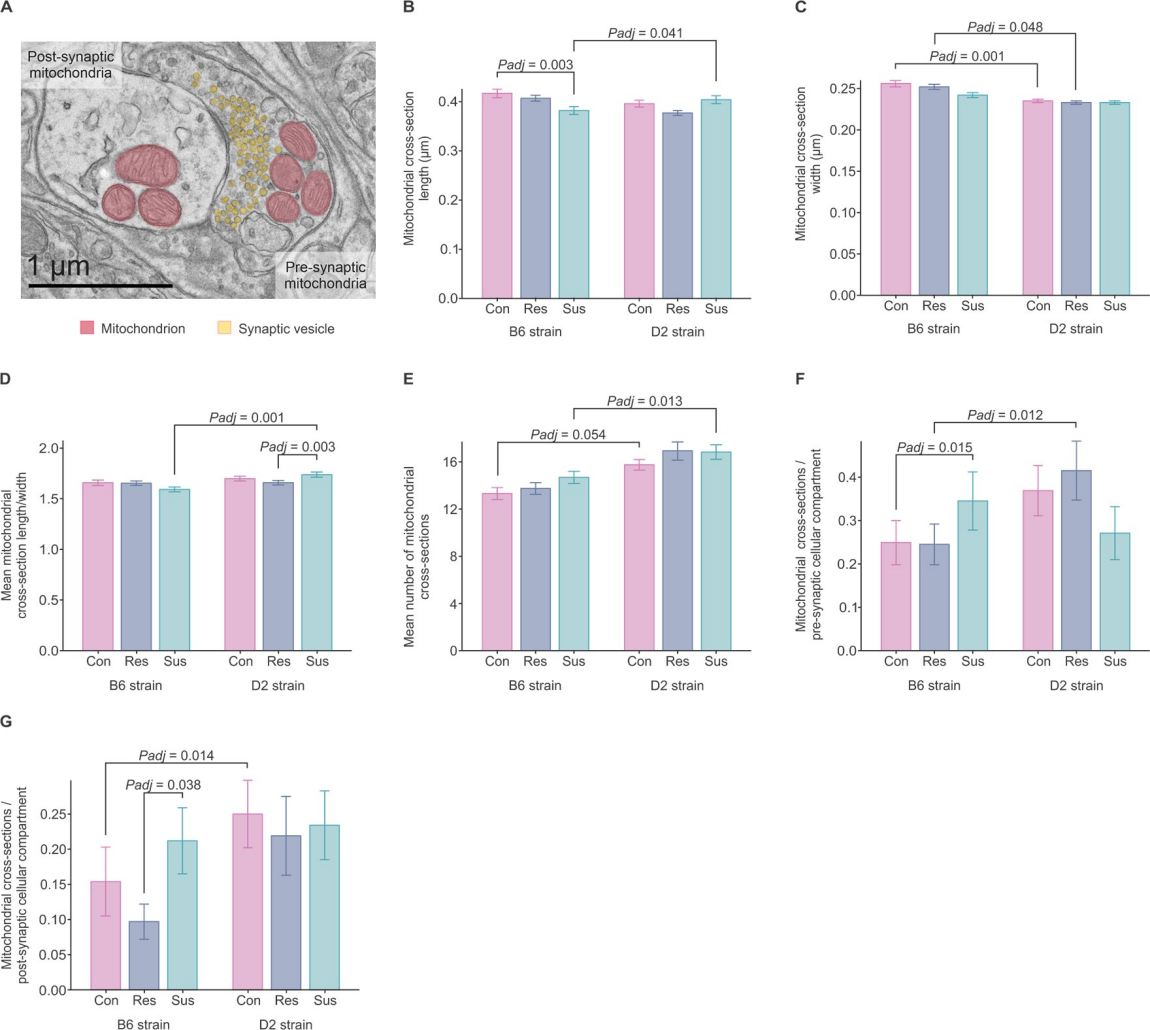
Chronic social defeat stress affects BNST mitochondrial morphology of B6 and D2 mice. (A) Transmission electron microscopy image showing pre- and post-synaptic neuronal compartments, mitochondria (red) and vesicles (yellow) located next to the synapse. (B-G) Graphs showing mean differences between B6 and D2 stress-susceptible, resilient and control mice in (B) maximum (length) and (C) minimum (width) diameters of mitochondrial cross-sections, (D) mean mitochondrial cross-section length/width ratio, i.e., maximum/minimum diameter (E) mean number of mitochondrial cross-sections (F-G) number of mitochondrial cross-sections per (F) pre-synaptic and (G) post-synaptic cellular compartment. *P*-values were adjusted for multiple testing with test-wise Bonferroni correction. Mean values ± S.E.M. B6: C57BL/6NCrl; Con: control; D2: DBA/2NCrl; Res: resilient; Sus: susceptible.

To determine if the observed differences in mitochondrial gene and protein expression are associated with changes in mitochondrial morphology in the pre-synaptic or post-synaptic compartments of neurons, we carried out transmission electron microscopy (TEM) in the BNST after CSDS in B6 and D2 mice. We classified mitochondria as “synaptic” if the synaptic density and vesicles within the post-synaptic terminal were clearly visible (Fig 8A). We observed stress-associated differences in maximum (length) and minimum (width) diameters of mitochondrial cross-sections in the B6 but not in the D2 strain. The B6 susceptible mice had on average 8.4% shorter mitochondrial cross-sections (maximum diameter) than B6 controls (*Padj* = 0.003) (Fig 8B), but no differences in the width (Fig 8C). However, the mean mitochondrial cross section length/width ratio in D2 susceptible, but not B6, mice was 5% larger than in the resilient group (*Padj* = 0.003) indicative of increased maximum, but decreased minimum, diameter (Fig 8D). The mean number of mitochondrial cross-sections was not influenced by stress (Fig 8E). However, when we investigated the pre-synaptic and post-synaptic compartments separately (Fig 8F and 8G), we detected 39% more pre-synaptic cross-sections in susceptible compared to the control B6 mice and 46% less post-synaptic cross-sections in resilient compared to the susceptible B6 mice. In addition, we observed significant strain differences. In general, B6 mice had wider diameter and smaller number of mitochondrial cross-sections than D2 mice, both pre- and post-synaptically (Fig 8B, 8F and 8G). Thus, consistently with our gene and protein differential expression data, we observed significant strain-dependent changes in mitochondrial morphology in the BNST following CSDS.

## Discussion

To identify the key biological pathways mediating resilience and susceptibility to psychosocial stress, a risk factor for onset and recurrence of anxiety disorders, we applied a cross-species multi-omics approach. In a chronic psychosocial stress mouse model, we found differential expression of mitochondrial-related genes and proteins both in the BNST and blood cells. However, the pattern of differential expression was opposite in the B6 and D2 mouse strains. Subsequently, we tested whether the same pathways are involved in acute anxiety provocation in panic disorder patients. Our analyses revealed a consistent convergence of differentially expressed mitochondria-related pathways in the blood samples from panic disorder patients after exposure-induced panic attack. As in the stress-susceptible D2 mice, these genes were downregulated during and after panic attack in patients. Consistently, we observed significant strain-dependent stress-associated differences in mitochondrial morphology in the BNST. Taken together, our results have uncovered an evolutionarily-conserved mitochondrial signature that characterizes anxiety-related behavior in mammals.

Of the mitochondrial genes, especially those related to oxidative phosphorylation were differentially expressed in both BNST and blood cells after chronic stress in mice and during exposure-induced panic attacks in panic disorder patients. These genes, that regulate both ATP production and apoptosis, had lower expression levels in the susceptible D2 mice compared to controls and in the panic disorder patients during and after panic attack. The expression levels of the same genes were higher in the susceptible B6 mice compared to controls. In a bidirectionally bred mouse model of trait anxiety (the HAB/LAB mice), we previously observed increased expression of electron transport chain proteins in the cingulate cortex synaptosomes of the high-anxiety mice [47]. In an outbred strain rat model of social behavior, highly anxious rats that were prone to become subordinate during a social encounter with a rat with low levels of anxiety had lower levels of mitochondrial complex I and II proteins in the nucleus accumbens [48]. In a study that specifically investigated gene expression of mtDNA-encoded genes [49], four of these genes (*mt-Nd1*, *mt-Nd3*, *mt-Nd6*, and *mt-Atp6*) were downregulated after acute immobilization stress in the hippocampus. However, after chronic immobilization stress *mt-Nd6* was upregulated. These effects were mediated by glucocorticoids. Thus, also previous studies have found changes in brain mitochondrial gene expression in anxiety-like behavior and after stress, but the directionality of these changes was depended on the model of anxiety and the duration of the applied stressor (e.g. acute versus chronic stress). Our results extend these previous observations by showing that the directionality of the changes may likely be influenced by the genetic background of the strain, and may be related to the innate anxiety level or stress susceptibility of these strains. Moreover, the greatest advantage of multi-omics studies is their ability to identify the most significantly affected pathways from measurements of thousands of molecules. Although mitochondrial pathways have previously been associated with anxiety, our hypothesis-free approach established their dysregulation as a major brain stress response.

We also observed differences in mitochondrial morphology and/or number of mitochondrial cross-sections in B6 and D2 mice after CSDS in the BNST. Stress-susceptible B6 mice had a larger number but shorter pre- and post-synaptic mitochondrial cross-sections compared to B6 control or resilient mice. In the D2 strain, stress-susceptible mice had slightly increased mitochondrial cross-section length/width ratio compared to the resilient mice. Previously, nocturnal aggression stress has been associated with slightly smaller pineal gland mitochondrial size in gerbils [50]. Chronic, but not acute, immobilization stress in rats leads to increased mitochondrial area in hippocampal mossy fiber terminals [51]. Thus, psychological stress is associated with morphological changes of mitochondria, but how these changes relate to mitochondrial function remains to be revealed. Cellular stress can affect mitochondrial morphology, e.g. in the form of hyperelongation, donut formation, or fragmentation in response to cytochrome c release [52]. In response to ER stress, which leads to downregulation of protein synthesis through the eIF2 pathway involved in mRNA translation, mitochondria reshape and become longer to promote cellular energy levels [53]. It has been proposed that the cumulative effect of stressors over time contribute to mitochondrial allostatic load and overload, and as a consequence lead to recalibration of mitochondrial structure and functional adaptation (e.g. activation of hormonal receptors) as well as dysregulation of gene expression, inflammatory response, and apoptosis [54–56]. In this model, mitochondria interact bidirectionally with stress mediators, but their adaptive capacity and the direction of changes related to mitochondrial function in response to stressors depend on the innate resilience of the organism and the effects of long-term programming during critical developmental periods.

In addition to the opposite directionality of mitochondria-related pathways, we found strain-specific expression patterns of differentially expressed miRNAs. Notably, miR-34c was expressed at a lower level in both blood cells and BNST of B6 resilient mice compared to controls after CSDS. Several members of the miRNA-34 family are differentially expressed in psychiatric conditions in humans: miR-34a is expressed at a higher levels in blood cells of patients with schizophrenia [57], and miR-34b-5p and mir-34c-5p in patients of major depressive disorder [58]. Furthermore, miR-34a expression is higher in the postmortem cerebellum of patients with bipolar disorder [59]. In mice, we have previously shown that both miR-34a and miR-34c are induced by acute and chronic stress in the amygdala, and that injection of miR-34c to amygdala is anxiolytic after a stress challenge [60]. In a rat model of early adolescent traumatic stress, miR-34c was upregulated in the hypothalamus [61]. Many of the miR-34 family effects on stress may be mediated through the CRFR1 [60]. We did not find CRFR1 differential expression in the BNST or blood, suggesting existence of alternative main targets in these tissues. In addition to these two miRNAs differentially expressed only in the B6 strain, we found two out of three miRNAs, miR-148a and miR-181b, to be DE in opposite directions between the strains in blood cells. miR-148a has been previously associated with panic disorder [45] and miR-181b has been identified as a possible marker for schizophrenia [46] and Alzheimer’s disease [62]. Higher expression of miR-181b has been previously correlated with an increase in mitochondrial oxidative stress and oxidative DNA damage [63], an early and systemic process in the pathophysiology of Alzheimer’s disease [64, 65]. Both miRNAs are highly conserved between mice and humans [66].

In addition to differential expression of mitochondria-related pathways and miRNAs, we found distinct molecular responses in the two strains in dopamine- and cAMP-regulated neuronal phosphoprotein (DARPP-32/PPP1R1B) and associated Dopamine-DARPP-32 Feedback in cAMP and Ca^2+^ Signaling pathway. Increased expression of DARPP-32 protein was previously observed in the prefrontal cortex and the amygdala of socially defeated B6 mice [36]. DARPP-32 has been proposed to act as an integrator of dopaminergic and glutamatergic signaling [67] and elevated levels of its truncated isoform were reported in schizophrenia, bipolar disorders and major depression as well as poor cognitive functioning [40]. These results implicate genetic background-dependent differences in susceptibility to chronic stress between the strains [68, 69]. Overall, our results suggest that transcriptomic response to stress is strongly dependent on the genetic background.

At the behavioral level, innately anxious D2 mice were more susceptible to CSDS than the less anxious B6 mice. While relatively few studies have examined strain differences in response to repeated stress [68, 70–72], and CSDS in particular [4, 73, 74], their findings similarly implicate heightened stress susceptibility in more anxious strains (e.g., BALB/cJ, D2). D2 resilient mice responded to CSDS with higher stress-induced locomotor activity in comparison to the D2 susceptible mice. Additionally, D2 resilient mice with higher social interaction ratios also had an elevated latency to immobility in the FST, suggesting resistance to behavioral despair or lack of adaptation aimed at energy conservation in the face of inescapable situation [75, 76]. In contrast, B6 mice did not differ in either measurement. The two strains also varied in the body weight development during and after CSDS. While the body weight decreased in the defeated D2 mice in comparison to the D2 controls and the baseline weight, the defeated B6 mice gained weight during the CSDS, similarly to the B6 controls. Taken together, we found that genetic background influences adaptation of different stress-coping strategies, as observed previously [4, 77].

For neuropsychiatric diseases, such as panic disorder, obtaining samples from the brain is essentially impossible for a reasonably large and representative sample sets. Therefore, the existence of tissue, which is more accessible and could be used as surrogate for gene expression in the central nervous system, is crucial. In our data set, the overall gene expression pattern between the BNST and blood cells was moderately correlated in both mouse strains. These results are in accordance with previous studies investigating gene expression similarities between the whole blood and multiple brain regions in humans, and suggest that gene expression is not perfectly correlated between brain and blood, but may be useful for studying certain pathways, e.g. related to translational control [78]. On the pathway level, oxidative phosphorylation-related genes were differentially expressed both in the mouse BNST and blood cells, and in the panic disorder patient blood cells. These genes were upregulated in the defeated B6 and downregulated in the defeated D2 mice compared to controls both in the BNST and blood cells. Strikingly, this pathway was downregulated in panic disorder patients directly and 24 h after an exposure-induced panic attack. It is interesting that the gene expression pattern of the patients resembles that of the more stress-susceptible mouse strain, suggesting that stress-susceptibility may involve a general modulation of genes associated with mitochondrial function. There are several caveats in comparing a post-mitotic brain region to a peripheral biospecimen, which are very different in terms of mitochondrial metabolism, but nonetheless, we observed a consistent and converging signature that warrants further investigations. Altogether, our data reinforce the utility of cross-species approaches in the identification of the biological basis of human anxiety disorders but advises careful selection of mouse strains for the best translatability of the findings.

Although we concentrated on mitochondrial pathways and anxiety susceptibility in our follow-up experiments, we also found several pathways in the mouse transcriptomic and proteomic experiments associated with stress resilience. Stress resilience is considered an active, adaptive response to stress, not merely a lack of maladaptive symptoms [79]. Studying of resilience is challenging in humans due to a lack of well-controlled cohorts. However, translational studies on stress resilience would be highly warranted to understand the underlying mechanisms providing putative means to enhance resilience in stress-susceptible individuals.

In conclusion, our cross-species multi–omics approach found a systemic evolutionarily conserved mitochondrial response in anxiety-related behaviors in mice and in panic disorder patients. We have produced a large amount of mRNA, miRNA, and protein expression data and made it publicly available to provide a resource for formulation of additional hypotheses on psychosocial stress-induced anxiety in mice, and panic disorder in humans. Our converging findings on stress-susceptibility in both brain and blood cells indicated dysregulation of translation and mitochondrial-related pathways. Further functional studies underlying the mechanisms behind the observed differences in mitochondrial morphology and the dysregulation of the mitochondria-related genes will provide much needed insight into the molecular basis of panic disorder and other anxiety disorders, a critical step in developing future targeted therapies.

## Materials and methods

### Ethics statements

All animal procedures were approved by the Regional State Administration Agency for Southern Finland (ESAVI-3801-041003-2011 and ESAVI/2766/04.10.07/2014) and carried out in accordance with directive 2010/63/EU of the European Parliament and of the Council, and the Finnish Act on the Protection of Animals Used for Science or Educational Purposes (497/2013). All human procedures and research were approved by the Ethics Committee of the Ludwig Maximilian University, Munich, Germany, Project MARS Anxiety, IRB Number 318/00, in accordance with the Declaration of Helsinki. Written informed consent was obtained from all participants.

### Animals

Male 5-week-old B6 and D2, and 13-26-week-old Clr-CD1 (CD-1) mice (Charles River Laboratories, Sulzfeld, Germany) were housed in pathogen-free, humidity- (50 ± 15%) and temperature-controlled (22 ± 2°C) animal facility on a 12 h light-dark cycle (lights on at 6 a.m.) and with ad libitum access to Teklad 2916 rodent chow (Envigo, Huntingdon, United Kingdom) and water. All B6 and D2 mice were acclimatized for 10 days prior to CSDS in groups of 4–6 mice housed in Makrolon Type III cages. CD-1 mice were acclimatized for one week in single individually ventilated cages (IVC) (Tecniplast, Buguggiate, Italy) prior to CD-1 aggressor screening.

### Behavioral experiments in mice

#### CD-1 aggressor screening

Before the beginning of the CSDS paradigm, all CD-1 mice were screened for appropriate aggression levels as previously described [3, 4, 80].

#### CSDS

B6 and D2 male mice were subjected to 10-day CSDS (Fig 1A) according to the original published protocol [80] with few modifications [4]. In brief, the protocol involved a daily confrontation of maximum of ten minutes of two conspecific mice, the resident (CD-1), also referred to as the aggressor, and the intruder (B6 or D2) (Fig 1B). The intruder mouse was rotated every day, during the 10 days of CSDS, to avoid habituation to the aggressors. The mean duration of the contact with an aggressor for the B6 strain was 6 min 5 s and for the D2 strain 3 min 15 s. During the 10-day protocol, the B6 and D2 control mice were housed in identical cages, separated by a clear plexiglass divider, with one mouse per side (Fig 1C) [80]. Similar to the defeated mice, every 24 hours the control mouse was placed into a cage with a new unfamiliar neighbor mouse. However, unlike the defeated mice, the control mice were always separated by a perforated plexiglass divider and were never in physical contact with their neighbors.

#### Social avoidance (SA) test

Twenty-four hours after the last day of CSDS, mice were exposed to social avoidance test (Fig 1D), as previously described [4, 6]. Briefly, the test consisted of two trials, each lasting 150 sec. During the first trial (*no target*), the defeated or control mouse was placed in the center of an open arena (42 cm x 42 cm) in which an empty perforated plexiglass cylinder was positioned next to one of the walls. During the second trial (*target*), the empty plexiglass cylinder was replaced with a new one containing an unfamiliar social *target* CD-1 mouse. The movements of the defeated or control mouse were tracked with a camera connected to a computer with EthoVision XT 10 software (Noldus Information Technology, Wageningen, Netherlands) and the amount of time the mouse spent in the interaction zone (IZ), that is the semicircle area around the plexiglass cylinder, was recorded. This test allowed us to divide the defeated mice into stress-susceptible and stress-resilient groups. To do so, we determined the mean and median social interaction (SI) ratio for a larger cohort of control mice separately in each strain (B6 = 126, D2 = 114) [4]. SI ratio was calculated by dividing the time spent in the IZ during the second trial by the time spent in the IZ during the first trial. Importantly, we confirmed that the total time of interaction with the aggressors during the CSDS did not correlate with the SI ratio of the defeated mice in neither the B6 nor the D2 strain (Pearson’s correlation, *r* = -0.034, *P* = 0.73 and *r* = -0.121, *P* = 0.32, respectively). Subsequently, we log-transformed the data from both defeated and control groups, to obtain normal distribution and removed outliers, defined as mice with SI ratio above > 3 IQRs from the median. We next divided the defeated mice to stress-resilient and susceptible groups based on the SI ratio, with the border determined as the controls’ mean SI score minus one SD. The SI score border value for B6 was 76.49, and for D2 105.99 [4]. Of the B6 mice subjected to SA test, 9% of the susceptible, 20% of the resilient and 35% of the control mice were included in our previous study [4]. The defeated and control mice for gene expression and proteomic profiling experiments (see below) were collected across six cohorts, with each cohort being equally divided between the experiments, and SI ratios being equally distributed between the experiments within each phenotypic group. We selected a subset of defeated and control animals for the sequencing and proteomics analyses based on their SI ratios. For the resilient and susceptible groups, we selected mice representing the phenotypic extremes, i.e., those with the highest and lowest SI ratios, respectively. For the control groups, we selected mice with SI ratios closest to the mean. We estimated the sample size based on successful prior sequencing experiments using the CSDS model [4, 16, 81]. However, for some groups (especially D2 resilient mice), we were limited by the availability of these mice, which could be obtained with reasonable number of defeated animals.

**Forced swim test (FST)** was performed five days after the end of CSDS as described in [4].

**Body weight** was recorded on days 1, 4, 6, 8, 10 and 12 of the CSDS. Following the exclusion of outliers, defined as a modified Z-score > 3.5 (*n* = B6: Resilient = 1, Control = 4; D2: Susceptible = 7, Resilient = 2, Control = 3) [82], mean percentage of original body weight was calculated as the difference between the first and fourth-12th days, divided by the first body weight. Inferential statistical testing by mixed-design repeated measure ANOVA with Tukey’s HSD multiple comparison test was carried out separately for both strains to assess the interaction effect on weight between time and phenotype. One-way ANOVA with Bonferroni correction was performed to estimate differences between all phenotypic groups, separately for each strain and day. All calculations were done with SPSS Statistics 25 (IBM, NY, USA).

### Gene expression profiling from mouse BNST and blood cells

#### Brain tissue collection

Mice were sacrificed by cervical dislocation 6–8 days after the end of the CSDS. The order, in which the mice were sacrificed, was counterbalanced across days and phenotypes. BNST was dissected as previously described [83]. Briefly, the brain was placed on a cold steel 1 mm brain matrix. A 2 mm slice was taken using designated anatomical coordinates, the optic chiasms, corresponding to Bregma -0.75–1.25 (Fig 2A). The slice was immediately frozen on dry ice and the area of interest below the lateral ventricles was punched using blunted 16G microdissecting needle (Sigma-Aldrich, MI, USA). The punches were flash-frozen in liquid nitrogen and stored at -80°C.

#### Blood collection

Trunk blood was collected at the moment of decapitation into a 1.3 ml Eppendorf tube coated with EDTA (Sarstedt, Vantaa, Finland). TRIzol LS reagent (Thermo Fisher Scientific, MA, USA) was immediately added (3:1 ratio of reagent to blood) and the samples were stored at -80°C for a maximum of two weeks prior to RNA extraction [84].

#### Immunoprecipitation of AGO2 protein

Immunoprecipitation of AGO2-bound miRNAs and their target mRNAs was performed as previously described [23].

#### Sequencing of RNA (RNA-seq), mRNA (mRNA-seq) and small RNA (miRNA-seq)

In total, five sequencing experiments were carried out (Fig 2C), three from the BNST, that is 1) sequencing of RNA, thereafter called data set A, and 2) sequencing of active miRNAs and their mRNA targets from the AGO2 immunoprecipitation samples, thereafter called data sets D and E, respectively, and two from blood cells, that is 3) sequencing of RNA and miRNAs extracted from blood cells, thereafter called data sets F and G, respectively. The data set A was previously included as a part of another publication [4]. Total RNA from the BNST and blood cells was extracted with TriReagent (Molecular Research Center Inc., OH, USA) or TRIzol LS reagent (Thermo Fisher Scientific, MA, USA), respectively, and RNA from the AGO2 immunoprecipitation samples was isolated using the RNeasy plus kit (Qiagen, Hilden, Germany) according to the manufacturers’ instructions. RNA quantity was measured using QuBit 2.0 Fluorometer (Invitrogen, CA, USA) and the quality assessed with an Agilent 2100 Bioanalyzer using RNA 600 Nano Chip and Small RNA Kits (Agilent Technologies, CA, USA).

For RNA-seq (data sets A and F), rRNA was depleted with custom Insert Dependent Adaptor Cleavage (InDA-C; data set A) primers or Ribozero Globin depletion Kit (data set F; Illumina, CA, USA) and fragmented using the S2 ultrasonicator (Covaris Inc., MA, USA). Sequencing libraries were prepared with Ovation Universal RNA-Seq System (data set A; NuGEN, Leek, Netherlands) or TruSeq Stranded mRNA Library Prep (data set E; Illumina, CA, USA) or TruSeq Stranded Total RNA (data set F; Illumina, CA, USA) Kits, size-selected with Pippin Prep (Sage Science, MA, USA) and sequenced on NextSeq 500 (single-end 86 bp, 84 bp and 75 bp respectively; Illumina, CA, USA). Following quality assesment with FastQC and adapter trimming with FastX toolkit v0.0.13, PCR duplicates were removed with PRINSEQ v0.20.4 (data set A). Reads were aligned with STAR v2.5.0c [85] using default settings to mouse genome GRCm38, and annotated to gene exons with HTSeq v0.6.1 [86] using GTF release 86 (update 2016–10).

For miRNA-seq (data sets D and G), sequencing libraries were prepared with TruSeq Small RNA Library Prep Kit (data set D; Illumina, CA, USA) and NEXTflex Small RNA-Seq Kit v3 (data set G; Bioo Scientific, TX, USA). The libraries were enriched by size selection with Pippin Prep (Sage Science, MA, USA) and sequenced on NextSeq 500 (single-end 76 bp; Illumina, CA, USA). Following adapter trimming with FastX toolkit v0.0.13 (data sets D and G) and PRINSEQ v0.20.4 (data set G), the reads were aligned to mouse reference miRNAs (miRBase v21) using miRDeep2 v0.0.7 [87] and to mouse genome GRCm38 (GRCm38) using bowtie v1.1.1 [88]. The expression levels of mature miRNAs were quantified using miRDeep2 v0.0.7 quantifier.pl module. We excluded one sample from data set D due to failed library preparation.

**Differential expression (DE) analysis** was performed using limma eBayes [24, 89] to identify statistically significantly DE genes between stress-resilient, susceptible, and same-strain controls. Low-abundance genes were removed prior to data normalization with voom [90], keeping genes with at least one count per million (CPM) in at least six (data sets A, F-G) or three (data sets D and E) samples [86]. Subsequently, the data was adjusted for library preparation batches (data set A), experimental cohort number (data set E) and detected surrogate variables (data set D) with ComBat [91]. Gene expression data is available under GEO accession GSE122840.

### Proteomic profiling from mouse BSNT

#### Brain tissue collection

Mice were anesthetized with isoflurane (4.0–4.2% for induction, 2.2–2.3% in 1 L per minute O2-flow for maintenance) and transcardially perfused with 0.9% saline solution (4°C). Following perfusion, the mice were decapitated and the BNST was collected as described earlier (Fig 2A).

#### Proteomic analysis

Four mg mouse BNST tissue was homogenized in 150 μl of protein extraction buffer (7 M Urea, 2 M Thiourea, 4% (v/w) CHAPS, 2% (v/w) ASB-14, 70 mM DTT) containing protease inhibitor cocktail tablets (Roche Diagnostics, Mannheim, Germany) and phosphatase inhibitor cocktails 1 and 2 (Sigma, MO, USA) using a pestle for 2 min and then sonicating in a water bath. The homogenized sample was centrifuged for 10 min at 20000 g at 4°C. The supernatant was collected, and the protein concentration was determined with a Bradford protein assay (Bio-Rad Laboratories GmbH, Munich, Germany). A reference sample was prepared from an equal mixture of all samples. One hundred μg protein samples were first reduced at 60°C for 30 min, and then alkylated by adding 50mM iodoacetamide and incubating for 30 min at RT in the dark. The samples were washed 3 times with 100 mM phosphate buffer, 1 M urea, pH 8.5 using Microcom UltraCel YM-30 filter (Millipore, MA USA), by spinning at 14000 g for 3 min at RT. Trypsin digestion was carried out using 1:50 enzyme to protein ratio with Trypsin sequencing Grade Modified (Serva Electrophoresis GmbH, Heidelberg, Germany), and incubating overnight at 37°C. The tryptic peptides were recovered by centrifugation at 15000 g x 10 min. Aliquots equivalent to 20 μg peptides were labeled with ICPL (Isotope Coded Protein Labeling) labeling reagents ICPL0 and ICPL6 (Serva Electrophoresis GmbH, Heidelberg, Germany), according to the manufacturer’s instruction. ICPL 6 labeled peptides were mixed with equal amounts of ICPL 0 labeled reference sample. The labeled peptide samples were desalted using OMIX tip (Varian, CA, USA). The desalted ICPL-labeled peptides were analyzed in triplicates by LC-MS/MS using Dionex HPLC system (Thermo Scientific, MA, USA) coupled to Q-Tof Impact HD II (Bruker Daltonic, Bremen, Germany) mass spectrometer. Two μg peptides were separated on an Acclaim PepMap RSLC nano column (C18, 50 cm x 75 μm) (Dionex, Thermo Scientific, MA USA) at 35°C, applying a 90 min gradient from 4 to 45% of 80% acetonitrile in 0.1% formic acid, at 300 nl/min flow rate. The mass spectrometer was run in positive mode in the mass range from 150 to 2200 m/z. Top 20 method was used to select the precursor ions for MS/MS. MS and MS/MS raw data were searched against SwissProt *Mus musculus* database using Mascot v2.3.0.2 (Matrix Science, London, UK) search engine implemented in ProteinScape v3.0 (Bruker Daltonik, Bremen, Germany). For the database search, carboamidomethylation on cysteine, ICPL 0 and ICPL 6 at lysine and aminoterminus were set as stable modifications; methionine oxidation was set as variable modification. Enzyme was set as trypsin, and one missed cleavage was allowed. The mass tolerance was set at 10 ppm for the precursor and 0.05 Da for the peptide fragments. The false discovery rate (*P*_*FDR*_) of 99% for all quantified peptides was determined through a target decoy approach. The H/L relative peptide quantification ratios were calculated based on the peptide pairs found in the MS spectra with the support of WRAPLC algorithm implemented in ProteinScape v3.0. Only proteins identified by at least two unique peptides in two out of three replicates, and with an H/L variability of less than 30% were selected for downstream analyses.

#### Differential expression (DE) analysis

For all selected proteins, an average ratio value across all technical replicates was calculated, log2-transformed and normalized for systematic biases with EigenMS [92] method implemented in DanteR R package [93]. The differential expression analysis was then performed using limma eBayes [24, 89, 94, 95]. Protein expression data is available at the Center for Computational Mass Spectrometry (MSV000083001).

### Human panic disorder sample

Twenty-one (*n* = 6 males, age 29.33 ± 8.48 years; *n* = 15 females, age 32.60 ± 9.61 years) non-medicated panic disorder patients were recruited in the anxiety disorder outpatient unit at the Max Planck Institute of Psychiatry, Munich, Germany. Panic disorder without (*n* = 3; 14.3%) or with (*n* = 18; 85.7%) comorbid agoraphobia was assigned as the primary diagnosis, mild secondary depression was allowed (*n* = 2; 9.5%). The diagnosis was ascertained by trained psychiatrists according to the Diagnostic and Statistical Manual of Mental Disorders (DSM)-IV criteria as previously described [96]. Exposure sessions were conducted outside the clinic, depending on the feared situation (e.g. subway, supermarket, tower) and specific concern (e.g. fainting, asphyxiation, losing control). The kind and site of exposure was determined by the patient, with the goal of highest fear provocation. On the day of the exposure and post-exposure day, the patients were instructed to eat a certain breakfast; smoking, exercises/sports or intake of caffeine was not allowed. Each exposure was performed in the morning, starting at 8 a.m. to 9 a.m. For all three time points (baseline, 1 h and 24 h post-exposure) peripheral blood was collected using PAXgene Blood RNA Tubes (PreAnalytiX, Hombrechtikon, Switzerland) and processed as previously described [96]. Blood cell RNA was hybridized to Illumina HumanHT-12 v4 Expression BeadChips (Illumina, CA, USA). Raw probe intensities were exported with Illumina’s GenomeStudio. Cross-hybridizing probes as well as probes binding to X and Y chromosomes were removed to avoid a possible gender effect [96]. Probes with detection *P*-value larger than 0.05 in > 50% of the samples were excluded from the analysis. For each transcript, normalization was performed using Variance stabilization and calibration for microarray data (VSN) R package. Subsequently, technical batches associated to Chip Barcode and Bead Chip ID were identified and removed with ComBat [91]. The probes were annotated to HGNC GeneSymbols using the Illumina platform annotation file and biomaRt R package v2.36.1 [97]. Probes not annotated to any genes and those annotated to multiple genes were excluded from downstream gene set enrichment analyses. Gene expression data is available in GEO (GSE119995).

#### Ingenuity Pathway Analysis (IPA)

The core and comparison network analyses were generated with IPA v.48207413 [27] for all genes (data set A) and proteins (data set B) with nominal *P* < 0.05 and absolute fold change (|FC|) **≥** 1.2. Analysis comparison is shown for dysregulated pathways with at least 2/12 comparisons with *P*_*FDR*_ < 0.05 and at least 4/12 comparisons with *P* < 0.05 (Fig 4A). In Fig 4B, we selected upstream regulators present in at least 25% of all comparisons with *P*_*FDR*_ < 0.05. Pathways and upstream regulators are organized alphabetically.

**Gene set enrichment analysis (GSEA)** was applied to all genes and proteins from mouse gene expression (data sets A and F) and proteomic experiments (data set B), as well as gene expression data from human PD cohort (data set H). All analyses were run using the Preranked tool implemented in GSEA Desktop v3.0 [43, 44] and the curated gene sets (C2) of the Molecular Signature Database (MSigDB) v6.0 (http://www.broad.mit.edu/gsea/). Metric rank for the analyzed genes and proteins was calculated as described before [98]. The significantly enriched gene sets present in at least 50% of the data sets, in their respective comparisons (S3 Fig, *P*_*FDR*_ < 0.25; and Fig 7D, *P*_*FDR*_ < 0.25), and both panic disorder (PD) patients’ blood data time points (Fig 7D), were selected for visualization. Gene sets are listed alphabetically. The DE genes, common between the strains in the susceptible mice in comparison to the controls (data sets A and F) were further investigated by hypergeometric distribution implemented in the MSigDB v6.0. The 100 most significant results with *P*_*FDR*_ < 0.05 were reported.

#### Gene Ontology (GO) term enrichment

We analyzed GO term enrichment for differentially expressed genes (*P* < 0.05 and |FC| **≥** 1.2) overlapping between the B6 and D2 susceptible mice in comparison to the controls using the topGO R package [99], with standard parameters. Classical enrichment analysis by testing over-representation of GO terms with the group of differentially expressed genes (*P* < 0.05) was used for the analysis of human data set (H). All expressed genes were used as a background.

### Western blot analysis

To select proteins for technical validation with Western blot, we performed a literature search on molecules overlapping the transcriptomic (A and E) and proteomic (B) data sets, and which were also present in at least one of the significantly dysregulated canonical pathways (Fig 4A) or mitochondria-related gene sets (S3 Fig), and found nine proteins (ADCY5, PPP1R1B, QDPR, GAD2, ATP2B1, PPP3CA, ATP6V1E1, GLUD1, and CYCS; Fig 5) [100]. We selected two, PPP1R1B and CYCS, which have been previously associated with psychiatric disorders [36–39], for validation. Validation samples included a subset of four or five samples used in LC-MS/MS analysis (data set B), with the exception of all D2 resilent mice being identical. Ten μg of total protein were separated by SDS-PAGE and transferred to Immobilon-FL membranes (Merck Millipore, MA, USA). After blocking with 5% non-fat milk in Tris-buffered saline with 0.1% Tween-20 (TBS/T), membranes were incubated overnight at 4°C with a primary antibody: mouse monoclonal anti-Cytochrome C antibody (1∶500, Santa Cruz Biotechnology, TX, USA, #sc13156) or mouse monoclonal anti-DARPP-32 antibody (1∶200, Santa Cruz Biotechnology #sc-271111). After washing, membranes were incubated with horseradish peroxidase (HRP)-conjugated secondary antibody (goat anti-rabbit, 1∶10000, Cell Signaling Technology, MA, USA, #7074S or goat anti-mouse, 1∶10000, Santa Cruz Biotechnology, #sc-516102). Signals were visualized with the Chemi Doc MP Imaging System (BioRad, Munich, Germany) after incubating membranes with enhanced chemiluminescence developing solution (Merck Millipore, Darmstadt, Germany). Expression levels of all proteins were normalized to Coomassie blue staining signals. Densitometric analyses were carried out using ImageJ software (National Institutes of Health, MD, USA). Statistical significance was calculated between data pairs with a one-tailed Student t test using Microsoft Excel.

### Transmission electron microscopy

#### Dissection

Brain sections (*n* = 3–4 mice per group) were prepared as previously described [4].

#### Imaging

Two ultrathin sections per mouse were imaged with Jem-1400 transmission electron microscope (Jeol, Tokyo, Japan) to collect 20 non-overlapping images (10 images/section) at x5000 magnification. We avoided taking images containing nerve tracts and selected those with only 1 to 3 cross-sections of myelinated fibers to ensure comparability between images.

#### Image analysis and quantification

The number of mitochondrial cross-sections and their size (length and width) were analyzed by a researcher blind to the sample group with the use of Microscopy Image Browser [101]. The analyzed image area consisted of 17.6 μm^2^. Only the mitochondrial cross-sections that fit entirely within the image area, which makes it possible to measure their maximum (length) and minimum (width) diameter, were counted. Synaptic densities were identified, and the number of mitochondrial cross-sections localized in the pre- or post-synaptic compartment was counted.

#### Statistical analysis

Every statistical group included 60 to 80 images from three to four animals. Due to the low number of animals per group, and to take into account within-subject dependencies of individual mitochondria measured from the same animal [102], we analyzed the group differences using generalized estimating equations. Pairwise contrasts were computed by Fisher’s LSD and corrected for multiple comparisons with the Bonferroni method. The statistical analysis was performed with SPSS Statistics 25 (IBM, NY, USA).

## Supporting information

S1 Fig Body weight during CSDS. We weighted all mice during CSDS and one day after the SA test. The percentage change in body weight from the baseline (day one) as a function of time is shown (mean ± 1 SEM). In the B6 strain, CSDS did not have a significant effect on body weight as all B6 mice gained weight throughout the duration of the experiment (mixed-design repeated measures ANOVA, F_5,157_ = 95.34, *P* = 9.45E^-46^). Additionally, no differences were observed between the susceptible or resilient mice in comparison to the same-strain controls on any day (one-way ANOVA with Bonferroni post hoc test, *Padj* > 0.134). Conversely, in the D2 strain, the body weight of all defeated animals decreased during CSDS (mixed-design repeated measures ANOVA, F_2,114_ = 41.793, *P* < 0.001). Although all defeated D2 mice weighted less than controls throughout (Bonferroni post hoc test, *Padj* < 0.010) and up to 48 h after the end of CSDS (Bonferroni post hoc test, *Padj* < 0.040), the weight of the stress-resilient and stress-susceptible mice did not differ on any day (one-way ANOVA with Bonferroni post hoc test, *Padj* = 1.000). *n* = B6: Susceptible = 34, Resilient = 77, Control = 55; D2: Susceptible = 55, Resilient = 6, Control = 59. Outlier criterion: modified Z-score > 3.5. Outliers: *n* = B6: Resilient = 1, Control = 4; D2: Susceptible = 7, Resilient = 2, Control = 3. *: *Padj* < 0.05, **: *Padj* < 0.01, ***: *Padj* < 0.001. B6: C57BL/6NCrl; D2: DBA/2NCrl.
(TIF)

S2 Fig Overlap of differentially expressed genes in the BNST after chronic social defeat stress. (A-B) Overlap of the differentially expressed (*P* < 0.05 and |FC| ≥ 1.2) genes between resilient versus control, susceptible versus control and susceptible versus resilient mice, separately in (A) B6 and (B) D2 strains. B6: C57BL/6NCrl; D2: DBA/2NCrl.
(TIF)

S3 Fig Integrated gene set enrichment analyses (GSEA) show dysregulation of mitochondria-related gene sets. Merged heatmap showing the most significant four overlapping gene sets between the transcriptomic (data set A) and proteomic (data set B) GSEA results in the BNST of CSDS mice. Only significant (*P*_*FDR*_ < 0.05) normalized enrichment scores (NES) are shown. Gene sets that did not pass the cut-off are marked in gray (NA). A positive (or negative) NES for a given gene set implicates its overrepresentation at the top (or bottom, respectively) of the ranked list of upregulated (or downregulated, respectively) genes. Gene sets are ordered alphabetically. B6: C57BL/6NCrl; BNST: bed nucleus of the stria terminalis; Con: control; D2: DBA/2NCrl; Res: resilient; Sus: susceptible.
(TIF)

S4 Fig **Correlation between PPP1R1B relative intensity in the BNST as detected by Western blot analysis and social interaction (SI) ratios in the (A) B6 strain and (B) D2 strain.** B6: C57BL/6NCrl; BNST: bed nucleus of the stria terminalis; D2: DBA/2NCrl; PPP1R1B: protein phosphatase 1 regulatory subunit 1B; *r*: Pearson correlation coefficient.
(TIF)

S1 Table The effect of chronic social defeat stress on behavior in B6 and D2 mice. All analyses performed with SPSS Statistics 25. B6: C57BL/6NCrl; D2: DBA/2NCrl; SA: social avoidance.
(XLSX)

S2 Table Number of differentially expressed mRNAs, miRNAs, and proteins after chronic social defeat stress. B6: C57BL/6NCrl; BNST: bed nucleus of stria terminalis; Con: control; D2: DBA/2NCrl; Res: resilient; Sus: susceptible.
(XLSX)

S3 Table 100 most significantly enriched gene sets (*P*_*FDR*_ < 0.05) within the differentially expressed genes (*P* < 0.05 and |FC| ≥ 1.2) overlapping between the B6 and D2 susceptible versus control comparisons in the BNST (data set A). Analysis performed with MSigDB v6.0 C2 curated gene sets. B6: C57BL/6NCrl; BNST: bed nucleus of the stria terminalis; D2: DBA/2NCrl.
(XLSX)

S4 Table Significantly enriched Gene Ontology (GO) terms (*P*_*FDR*_ < 0.05) within the differentially expressed genes (*P* < 0.05 and |FC| ≥ 1.2) overlapping between the B6 and D2 susceptible versus control comparisons in the BNST (data set A). Analysis performed with topGO R package. B6: C57BL/6NCrl; BNST: bed nucleus of the stria terminalis; D2: DBA/2NCrl; GO: Gene Ontology.
(XLSX)

S5 Table Ingenuity Pathway Analysis (IPA) miRNA Target Filter results of identified differentially expressed (*P* < 0.05 and |FC| ≥ 1.2) active miRNA-mRNA pairs (data sets D and E) from AGO2 immunoprecipitation-sequencing in the BNST after chronic social defeat stress. B6: C57BL/6NCrl; BNST: bed nucleus of the stria terminalis; FC: fold change; IPA: Ingenuity Pathway Analysis.
(XLSX)

S6 Table Overlap between statistically significant differentially expressed (*P* < 0.05 and |FC| ≥ 1.2) transcriptomic (data sets A and E) and proteomic (data set B) data. Data generated from mouse BNST following exposure to chronic social defeat stress. All non-significant results (*P* > 0.05 and |FC| < 1.2) are indicated by a hyphen ("-"). B6: C57BL/6NCrl; D2: DBA/2NCrl; FC: fold change.
(XLSX)

S7 Table 100 most significantly enriched gene sets (*P*_*FDR*_ < 0.05) within the differentially expressed genes (*P* < 0.05 and |FC| ≥ 1.2) overlapping between the B6 and D2 susceptible versus control comparisons in the blood cells (data set F). Analysis performed with MSigDB v6.0 C2 curated gene sets. B6: C57BL/6NCrl; BNST: bed nucleus of the stria terminalis; D2: DBA/2NCrl.
(XLSX)

S8 Table Significantly enriched Gene Ontology (GO) terms (*P*_*FDR*_ < 0.05) within the differentially expressed genes (*P* < 0.05 and |FC| ≥ 1.2) overlapping between the B6 and D2 susceptible versus control comparisons in the blood cells (data set F). Analysis performed with topGO R package. B6: C57BL/6NCrl; BNST: bed nucleus of the stria terminalis; D2: DBA/2NCrl; GO: Gene Ontology.
(XLSX)

S9 Table Differentially expressed (*P* < 0.05 and |FC| ≥ 1.2) miRNA in blood cells of B6 and D2 mice subjected to chronic social defeat stress (data set G). All non-significant results (*P* > 0.05 and |FC| < 1.2) are indicated by a hyphen ("-"). B6: C57BL/6NCrl; D2: DBA/2NCrl; FC: fold change.
(XLSX)

S10 Table Significantly overrepresented Gene Ontology (GO) terms (*P*_*FDR*_ < 0.05) within the differentially expressed genes (*P* < 0.05) in panic disorder (PD) patients’ blood cells (data set H) collected directly and 24 h after exposure-induced panic attack in comparison to baseline measurement (0 h and 24 h, respectively). Analysis performed with topGO R package. GO: Gene Ontology.
(XLSX)

## References

[1] Pirkola S, Isometsa E, Aro H, Kestila L, Hamalainen J, Veijola J, et al Childhood adversities as risk factors for adult mental disorders: results from the Health 2000 study. Soc Psychiatry Psychiatr Epidemiol. 2005;40(10):769–77. 10.1007/s00127-005-0950-x 16205853

[2] Nestler EJ, Hyman SE. Animal models of neuropsychiatric disorders. Nat Neurosci. 2010;13(10):1161–9. 10.1038/nn.2647 20877280 PMC3750731

[3] Laine MA, Sokolowska E, Dudek M, Callan SA, Hyytia P, Hovatta I. Brain activation induced by chronic psychosocial stress in mice. Sci Rep. 2017;7(1):15061 10.1038/s41598-017-15422-5 29118417 PMC5678090

[4] Laine MA, Trontti K, Misiewicz Z, Sokolowska E, Kulesskaya N, Heikkinen A, et al Genetic Control of Myelin Plasticity after Chronic Psychosocial Stress. eNEURO. 2018;5(4): ENEURO.0166-18.2018 10.1523/ENEURO.0166-18.2018 30073192 PMC6071195

[5] Avgustinovich DF, Kovalenko IL, Kudryavtseva NN. A model of anxious depression: persistence of behavioral pathology. Neurosci Behav Physiol. 2005;35(9):917–24. 10.1007/s11055-005-0146-6 16270173

[6] Krishnan V, Han MH, Graham DL, Berton O, Renthal W, Russo SJ, et al Molecular adaptations underlying susceptibility and resistance to social defeat in brain reward regions. Cell. 2007;131(2):391–404. 10.1016/j.cell.2007.09.018 17956738

[7] Wittchen HU, Jacobi F, Rehm J, Gustavsson A, Svensson M, Jonsson B, et al The size and burden of mental disorders and other disorders of the brain in Europe 2010. Eur Neuropsychopharmacol. 2011;21(9):655–79. 10.1016/j.euroneuro.2011.07.018 21896369

[8] Craske MG, Stein MB, Eley TC, Milad MR, Holmes A, Rapee RM, et al Anxiety disorders. Nat Rev Dis Primers. 2017;3:17024 10.1038/nrdp.2017.24 28470168 PMC11009418

[9] Ising M, Hohne N, Siebertz A, Parchmann AM, Erhardt A, Keck M. Stress response regulation in panic disorder. Curr Pharm Des. 2012;18(35):5675–84. 10.2174/138161212803530880 22632473

[10] Faravelli C, Lo Sauro C, Godini L, Lelli L, Benni L, Pietrini F, et al Childhood stressful events, HPA axis and anxiety disorders. World J Psychiatry. 2012;2(1):13–25. 10.5498/wjp.v2.i1.13 24175164 PMC3782172

[11] Klauke B, Deckert J, Reif A, Pauli P, Domschke K. Life events in panic disorder-an update on "candidate stressors". Depress Anxiety. 2010;27(8):716–30. 10.1002/da.20667 20112245

[12] Faravelli C, Lo Sauro C, Lelli L, Pietrini F, Lazzeretti L, Godini L, et al The role of life events and HPA axis in anxiety disorders: a review. Curr Pharm Des. 2012;18(35):5663–74. 10.2174/138161212803530907 22632471

[13] Provencal N, Binder EB. The effects of early life stress on the epigenome: From the womb to adulthood and even before. Exp Neurol. 2015;268:10–20. 10.1016/j.expneurol.2014.09.001 25218020

[14] Sokolowska E, Hovatta I. Anxiety genetics—findings from cross-species genome-wide approaches. Biol Mood Anxiety Disord. 2013;3(1):9 10.1186/2045-5380-3-9 23659354 PMC3655048

[15] Floriou-Servou A, von Ziegler L, Stalder L, Sturman O, Privitera M, Rassi A, et al Distinct Proteomic, Transcriptomic, and Epigenetic Stress Responses in Dorsal and Ventral Hippocampus. Biol Psychiatry. 2018;84(7):531–41. 10.1016/j.biopsych.2018.02.003 29605177

[16] Bagot RC, Parise EM, Pena CJ, Zhang HX, Maze I, Chaudhury D, et al Ventral hippocampal afferents to the nucleus accumbens regulate susceptibility to depression. Nat Commun. 2015;6:7062 10.1038/ncomms8062 25952660 PMC4430111

[17] Lebow MA, Chen A. Overshadowed by the amygdala: the bed nucleus of the stria terminalis emerges as key to psychiatric disorders. Mol Psychiatry. 2016;21(4):450–63. 10.1038/mp.2016.1 26878891 PMC4804181

[18] Gungor NZ, Pare D. Functional Heterogeneity in the Bed Nucleus of the Stria Terminalis. J Neurosci. 2016;36(31):8038–49. 10.1523/JNEUROSCI.0856-16.2016 27488624 PMC4971356

[19] Hovatta I, Tennant RS, Helton R, Marr RA, Singer O, Redwine JM, et al Glyoxalase 1 and glutathione reductase 1 regulate anxiety in mice. Nature. 2005;438(7068):662–6. 10.1038/nature04250 16244648

[20] Pleil KE, Lopez A, McCall N, Jijon AM, Bravo JP, Kash TL. Chronic stress alters neuropeptide Y signaling in the bed nucleus of the stria terminalis in DBA/2J but not C57BL/6J mice. Neuropharmacology. 2012;62(4):1777–86. 10.1016/j.neuropharm.2011.12.002 22182779 PMC3269561

[21] Anyan J, Amir S. Too Depressed to Swim or Too Afraid to Stop? A Reinterpretation of the Forced Swim Test as a Measure of Anxiety-Like Behavior. Neuropsychopharmacology. 2018;43(5):931–3. 10.1038/npp.2017.260 29210364 PMC5854810

[22] Commons KG, Cholanians AB, Babb JA, Ehlinger DG. The Rodent Forced Swim Test Measures Stress-Coping Strategy, Not Depression-like Behavior. ACS Chem Neurosci. 2017;8(5):955–60. 10.1021/acschemneuro.7b00042 28287253 PMC5518600

[23] Volk N, Paul ED, Haramati S, Eitan C, Fields BK, Zwang R, et al MicroRNA-19b associates with Ago2 in the amygdala following chronic stress and regulates the adrenergic receptor beta 1. J Neurosci. 2014;34(45):15070–82. 10.1523/JNEUROSCI.0855-14.2014 25378171 PMC6608371

[24] Smyth GK. Linear models and empirical bayes methods for assessing differential expression in microarray experiments. Stat Appl Genet Mol Biol. 2004;3:Article3 10.2202/1544-6115.1027 16646809

[25] Volk N, Pape JC, Engel M, Zannas AS, Cattane N, Cattaneo A, et al Amygdalar MicroRNA-15a Is Essential for Coping with Chronic Stress. Cell Rep. 2016;17(7):1882–91. 10.1016/j.celrep.2016.10.038 27829158 PMC5120368

[26] Leung AK, Sharp PA. MicroRNA functions in stress responses. Mol Cell. 2010;40(2):205–15. 10.1016/j.molcel.2010.09.027 20965416 PMC2996264

[27] Qiagen Inc. Ingenuity Pathway Analysis 2018 [cited 25 January 2019] [Internet]. Available from: https://www.qiagenbioinformatics.com/products/ingenuity-pathway-analysis/.

[28] Urbanska M, Gozdz A, Swiech LJ, Jaworski J. Mammalian target of rapamycin complex 1 (mTORC1) and 2 (mTORC2) control the dendritic arbor morphology of hippocampal neurons. J Biol Chem. 2012;287(36):30240–56. 10.1074/jbc.M112.374405 22810227 PMC3436277

[29] Skalecka A, Liszewska E, Bilinski R, Gkogkas C, Khoutorsky A, Malik AR, et al mTOR kinase is needed for the development and stabilization of dendritic arbors in newly born olfactory bulb neurons. Dev Neurobiol. 2016;76(12):1308–27. 10.1002/dneu.22392 27008592 PMC5132010

[30] Siuta MA, Robertson SD, Kocalis H, Saunders C, Gresch PJ, Khatri V, et al Dysregulation of the norepinephrine transporter sustains cortical hypodopaminergia and schizophrenia-like behaviors in neuronal rictor null mice. PLoS Biol. 2010;8(6):e1000393 10.1371/journal.pbio.1000393 20543991 PMC2882427

[31] Ruiz-Perez MV, Henley AB, Arsenian-Henriksson M. The MYCN Protein in Health and Disease. Genes (Basel). 2017;8(4). 10.3390/genes8040113 PMC5406860 28358317

[32] Jacobs EG, Holsen LM, Lancaster K, Makris N, Whitfield-Gabrieli S, Remington A, et al 17beta-estradiol differentially regulates stress circuitry activity in healthy and depressed women. Neuropsychopharmacology. 2015;40(3):566–76. 10.1038/npp.2014.203 25113601 PMC4289944

[33] Karagkouni D, Paraskevopoulou MD, Chatzopoulos S, Vlachos IS, Tastsoglou S, Kanellos I, et al DIANA-TarBase v8: a decade-long collection of experimentally supported miRNA-gene interactions. Nucleic Acids Res. 2018;46(D1):D239–D45. 10.1093/nar/gkx1141 29156006 PMC5753203

[34] Xiao F, Zuo Z, Cai G, Kang S, Gao X, Li T. miRecords: an integrated resource for microRNA-target interactions. Nucleic Acids Res. 2009;37:D105–10. 10.1093/nar/gkn851 18996891 PMC2686554

[35] Agarwal V, Bell GW, Nam JW, Bartel DP. Predicting effective microRNA target sites in mammalian mRNAs. Elife. 2015;4. 10.7554/eLife.05005 PMC4532895 26267216

[36] Jin HM, Shrestha Muna S, Bagalkot TR, Cui Y, Yadav BK, Chung YC. The effects of social defeat on behavior and dopaminergic markers in mice. Neuroscience. 2015;288:167–77. 10.1016/j.neuroscience.2014.12.043 25575945

[37] Kovalenko IL, Smagin DA, Galyamina AG, Orlov YL, Kudryavtseva NN. [Changes in the Expression of Dopaminergic Genes in Brain Structures of Male Mice Exposed to Chronic Social Defeat Stress: An RNA-seq Study]. Mol Biol (Mosk). 2016;50(1):184–7. 27028825 10.7868/S0026898416010080

[38] Scaini G, Fries GR, Valvassori SS, Zeni CP, Zunta-Soares G, Berk M, et al Perturbations in the apoptotic pathway and mitochondrial network dynamics in peripheral blood mononuclear cells from bipolar disorder patients. Transl Psychiatry. 2017;7(5):e1111 10.1038/tp.2017.83 28463235 PMC5534951

[39] Hroudova J, Fisar Z. Connectivity between mitochondrial functions and psychiatric disorders. Psychiatry Clin Neurosci. 2011;65(2):130–41. 10.1111/j.1440-1819.2010.02178.x 21414088

[40] Kunii Y, Hyde TM, Ye T, Li C, Kolachana B, Dickinson D, et al Revisiting DARPP-32 in postmortem human brain: changes in schizophrenia and bipolar disorder and genetic associations with t-DARPP-32 expression. Mol Psychiatry. 2014;19(2):192–9. 10.1038/mp.2012.174 23295814

[41] Clay HB, Sillivan S, Konradi C. Mitochondrial dysfunction and pathology in bipolar disorder and schizophrenia. Int J Dev Neurosci. 2011;29(3):311–24. 10.1016/j.ijdevneu.2010.08.007 20833242 PMC3010320

[42] Youle RJ, Karbowski M. Mitochondrial fission in apoptosis. Nat Rev Mol Cell Biol. 2005;6(8):657–63. 10.1038/nrm1697 16025099

[43] Subramanian A, Tamayo P, Mootha VK, Mukherjee S, Ebert BL, Gillette MA, et al Gene set enrichment analysis: a knowledge-based approach for interpreting genome-wide expression profiles. Proc Natl Acad Sci U S A. 2005;102(43):15545–50. 10.1073/pnas.0506580102 16199517 PMC1239896

[44] Mootha VK, Lindgren CM, Eriksson KF, Subramanian A, Sihag S, Lehar J, et al PGC-1alpha-responsive genes involved in oxidative phosphorylation are coordinately downregulated in human diabetes. Nat Genet. 2003;34(3):267–73. 10.1038/ng1180 12808457

[45] Muinos-Gimeno M, Espinosa-Parrilla Y, Guidi M, Kagerbauer B, Sipila T, Maron E, et al Human microRNAs miR-22, miR-138-2, miR-148a, and miR-488 are associated with panic disorder and regulate several anxiety candidate genes and related pathways. Biol Psychiatry. 2011;69(6):526–33. 10.1016/j.biopsych.2010.10.010 21168126

[46] He K, Guo C, He L, Shi Y. MiRNAs of peripheral blood as the biomarker of schizophrenia. Hereditas. 2018;155:9 10.1186/s41065-017-0044-2 28860957 PMC5575894

[47] Filiou MD, Zhang Y, Teplytska L, Reckow S, Gormanns P, Maccarrone G, et al Proteomics and metabolomics analysis of a trait anxiety mouse model reveals divergent mitochondrial pathways. Biol Psychiatry. 2011;70(11):1074–82. 10.1016/j.biopsych.2011.06.009 21791337

[48] Hollis F, van der Kooij MA, Zanoletti O, Lozano L, Canto C, Sandi C. Mitochondrial function in the brain links anxiety with social subordination. Proc Natl Acad Sci U S A. 2015;112(50):15486–91. 10.1073/pnas.1512653112 26621716 PMC4687564

[49] Hunter RG, Seligsohn M, Rubin TG, Griffiths BB, Ozdemir Y, Pfaff DW, et al Stress and corticosteroids regulate rat hippocampal mitochondrial DNA gene expression via the glucocorticoid receptor. Proc Natl Acad Sci U S A. 2016;113(32):9099–104. 10.1073/pnas.1602185113 27457949 PMC4987818

[50] Heinzeller T. Impact of psychosocial stress on pineal structure of male gerbils (Meriones unguiculatus, cricetidae). J Pineal Res. 1985;2(2):145–59. 3831304 10.1111/j.1600-079x.1985.tb00635.x

[51] Magarinos AM, Verdugo JM, McEwen BS. Chronic stress alters synaptic terminal structure in hippocampus. Proc Natl Acad Sci U S A. 1997;94(25):14002–8. 10.1073/pnas.94.25.14002 9391142 PMC28422

[52] Eisner V, Picard M, Hajnoczky G. Mitochondrial dynamics in adaptive and maladaptive cellular stress responses. Nat Cell Biol. 2018;20(7):755–65. 10.1038/s41556-018-0133-0 29950571 PMC6716149

[53] Lebeau J, Saunders JM, Moraes VWR, Madhavan A, Madrazo N, Anthony MC, et al The PERK Arm of the Unfolded Protein Response Regulates Mitochondrial Morphology during Acute Endoplasmic Reticulum Stress. Cell Rep. 2018;22(11):2827–36. 10.1016/j.celrep.2018.02.055 29539413 PMC5870888

[54] Picard M, McEwen BS. Psychological Stress and Mitochondria: A Systematic Review. Psychosom Med. 2018;80(2):141–53. 10.1097/PSY.0000000000000545 29389736 PMC5901654

[55] Picard M, McEwen BS. Psychological Stress and Mitochondria: A Conceptual Framework. Psychosom Med. 2018;80(2):126–40. 10.1097/PSY.0000000000000544 29389735 PMC5901651

[56] Picard M, McEwen BS, Epel ES, Sandi C. An energetic view of stress: Focus on mitochondria. Front Neuroendocrinol. 2018. 10.1016/j.yfrne.2018.01.001 PMC5964020 29339091

[57] Lai CY, Yu SL, Hsieh MH, Chen CH, Chen HY, Wen CC, et al MicroRNA expression aberration as potential peripheral blood biomarkers for schizophrenia. PLoS One. 2011;6(6):e21635 10.1371/journal.pone.0021635 21738743 PMC3126851

[58] Sun N, Lei L, Wang Y, Yang C, Liu Z, Li X, et al Preliminary comparison of plasma notch-associated microRNA-34b and -34c levels in drug naive, first episode depressed patients and healthy controls. J Affect Disord. 2016;194:109–14. 10.1016/j.jad.2016.01.017 26807671

[59] Bavamian S, Mellios N, Lalonde J, Fass DM, Wang J, Sheridan SD, et al Dysregulation of miR-34a links neuronal development to genetic risk factors for bipolar disorder. Mol Psychiatry. 2015;20(5):573–84. 10.1038/mp.2014.176 25623948 PMC4414679

[60] Haramati S, Navon I, Issler O, Ezra-Nevo G, Gil S, Zwang R, et al MicroRNA as repressors of stress-induced anxiety: the case of amygdalar miR-34. J Neurosci. 2011;31(40):14191–203. 10.1523/JNEUROSCI.1673-11.2011 21976504 PMC6623664

[61] Li C, Liu Y, Liu D, Jiang H, Pan F. Dynamic Alterations of miR-34c Expression in the Hypothalamus of Male Rats after Early Adolescent Traumatic Stress. Neural Plast. 2016;2016:5249893 10.1155/2016/5249893 26925271 PMC4746392

[62] Femminella GD, Ferrara N, Rengo G. The emerging role of microRNAs in Alzheimer's disease. Front Physiol. 2015;6:40 10.3389/fphys.2015.00040 25729367 PMC4325581

[63] Schipper HM, Maes OC, Chertkow HM, Wang E. MicroRNA expression in Alzheimer blood mononuclear cells. Gene Regul Syst Bio. 2007;1:263–74. 19936094 10.4137/grsb.s361 PMC2759133

[64] Migliore L, Fontana I, Trippi F, Colognato R, Coppede F, Tognoni G, et al Oxidative DNA damage in peripheral leukocytes of mild cognitive impairment and AD patients. Neurobiol Aging. 2005;26(5):567–73. 10.1016/j.neurobiolaging.2004.07.016 15708428

[65] Bhatnagar S, Chertkow H, Schipper HM, Yuan Z, Shetty V, Jenkins S, et al Increased microRNA-34c abundance in Alzheimer's disease circulating blood plasma. Front Mol Neurosci. 2014;7:2 10.3389/fnmol.2014.00002 24550773 PMC3912349

[66] Kiezun A, Artzi S, Modai S, Volk N, Isakov O, Shomron N. miRviewer: a multispecies microRNA homologous viewer. BMC Res Notes. 2012;5:92 10.1186/1756-0500-5-92 22330228 PMC3292992

[67] Fernandez E, Schiappa R, Girault JA, Le Novere N. DARPP-32 is a robust integrator of dopamine and glutamate signals. PLoS Comput Biol. 2006;2(12):e176 10.1371/journal.pcbi.0020176 17194217 PMC1761654

[68] Mozhui K, Karlsson RM, Kash TL, Ihne J, Norcross M, Patel S, et al Strain differences in stress responsivity are associated with divergent amygdala gene expression and glutamate-mediated neuronal excitability. J Neurosci. 2010;30(15):5357–67. 10.1523/JNEUROSCI.5017-09.2010 20392957 PMC2866495

[69] Malki K, Mineur YS, Tosto MG, Campbell J, Karia P, Jumabhoy I, et al Pervasive and opposing effects of Unpredictable Chronic Mild Stress (UCMS) on hippocampal gene expression in BALB/cJ and C57BL/6J mouse strains. BMC Genomics. 2015;16:262 10.1186/s12864-015-1431-6 25879669 PMC4412144

[70] Pothion S, Bizot JC, Trovero F, Belzung C. Strain differences in sucrose preference and in the consequences of unpredictable chronic mild stress. Behav Brain Res. 2004;155(1):135–46. 10.1016/j.bbr.2004.04.008 15325787

[71] Anisman H, Matheson K. Stress, depression, and anhedonia: caveats concerning animal models. Neurosci Biobehav Rev. 2005;29(4–5):525–46. 10.1016/j.neubiorev.2005.03.007 15925696

[72] Mineur YS, Belzung C, Crusio WE. Effects of unpredictable chronic mild stress on anxiety and depression-like behavior in mice. Behav Brain Res. 2006;175(1):43–50. 10.1016/j.bbr.2006.07.029 17023061

[73] Razzoli M, Domenici E, Carboni L, Rantamaki T, Lindholm J, Castren E, et al A role for BDNF/TrkB signaling in behavioral and physiological consequences of social defeat stress. Genes Brain Behav. 2011;10(4):424–33. 10.1111/j.1601-183X.2011.00681.x 21272243

[74] Savignac HM, Dinan TG, Cryan JF. Resistance to early-life stress in mice: effects of genetic background and stress duration. Front Behav Neurosci. 2011;5:13 10.3389/fnbeh.2011.00013 21519375 PMC3075880

[75] Arai I, Tsuyuki Y, Shiomoto H, Satoh M, Otomo S. Decreased body temperature dependent appearance of behavioral despair in the forced swimming test in mice. Pharmacol Res. 2000;42(2):171–6. 10.1006/phrs.2000.0672 10887048

[76] Petit-Demouliere B, Chenu F, Bourin M. Forced swimming test in mice: a review of antidepressant activity. Psychopharmacology (Berl). 2005;177(3):245–55. 15609067 10.1007/s00213-004-2048-7

[77] Kulesskaya N, Karpova NN, Ma L, Tian L, Voikar V. Mixed housing with DBA/2 mice induces stress in C57BL/6 mice: implications for interventions based on social enrichment. Front Behav Neurosci. 2014;8:257 10.3389/fnbeh.2014.00257 25147512 PMC4123727

[78] Sullivan PF, Fan C, Perou CM. Evaluating the comparability of gene expression in blood and brain. Am J Med Genet B Neuropsychiatr Genet. 2006;141B(3):261–8. 10.1002/ajmg.b.30272 16526044

[79] Russo SJ, Murrough JW, Han MH, Charney DS, Nestler EJ. Neurobiology of resilience. Nat Neurosci. 2012;15(11):1475–84. 10.1038/nn.3234 23064380 PMC3580862

[80] Golden SA, Covington HE 3rd, Berton O, Russo SJ. A standardized protocol for repeated social defeat stress in mice. Nat Protoc. 2011;6(8):1183–91. 10.1038/nprot.2011.361 21799487 PMC3220278

[81] Lorsch ZS, Loh YE, Purushothaman I, Walker DM, Parise EM, Salery M, et al Estrogen receptor alpha drives pro-resilient transcription in mouse models of depression. Nat Commun. 2018;9(1):1116 10.1038/s41467-018-03567-4 29549264 PMC5856766

[82] Iglewicz B, Hoaglin DC. How to detect and handle outliers. Milwaukee, Wis: ASQC Quality Press; 1993 pp. 16

[83] Lebow M, Neufeld-Cohen A, Kuperman Y, Tsoory M, Gil S, Chen A. Susceptibility to PTSD-like behavior is mediated by corticotropin-releasing factor receptor type 2 levels in the bed nucleus of the stria terminalis. J Neurosci. 2012;32(20):6906–16. 10.1523/JNEUROSCI.4012-11.2012 22593059 PMC6622202

[84] Winn ME, Zapala MA, Hovatta I, Risbrough VB, Lillie E, Schork NJ. The effects of globin on microarray-based gene expression analysis of mouse blood. Mamm Genome. 2010;21(5–6):268–75. 10.1007/s00335-010-9261-y 20473674 PMC2890980

[85] Dobin A, Davis CA, Schlesinger F, Drenkow J, Zaleski C, Jha S, et al STAR: ultrafast universal RNA-seq aligner. Bioinformatics. 2013;29(1):15–21. 10.1093/bioinformatics/bts635 23104886 PMC3530905

[86] Anders S, Pyl PT, Huber W. HTSeq—a Python framework to work with high-throughput sequencing data. Bioinformatics. 2015;31(2):166–9. 10.1093/bioinformatics/btu638 25260700 PMC4287950

[87] Friedlander MR, Chen W, Adamidi C, Maaskola J, Einspanier R, Knespel S, et al Discovering microRNAs from deep sequencing data using miRDeep. Nat Biotechnol. 2008;26(4):407–15. 10.1038/nbt1394 18392026

[88] Langmead B, Trapnell C, Pop M, Salzberg SL. Ultrafast and memory-efficient alignment of short DNA sequences to the human genome. Genome Biol. 2009;10(3):R25 10.1186/gb-2009-10-3-r25 19261174 PMC2690996

[89] Phipson B, Lee S, Majewski IJ, Alexander WS, Smyth GK. Robust Hyperparameter Estimation Protects against Hypervariable Genes and Improves Power to Detect Differential Expression. Ann Appl Stat. 2016;10(2):946–63. 10.1214/16-AOAS920 28367255 PMC5373812

[90] Law CW, Chen Y, Shi W, Smyth GK. voom: Precision weights unlock linear model analysis tools for RNA-seq read counts. Genome Biol. 2014;15(2):R29 10.1186/gb-2014-15-2-r29 24485249 PMC4053721

[91] Johnson WE, Li C, Rabinovic A. Adjusting batch effects in microarray expression data using empirical Bayes methods. Biostatistics. 2007;8(1):118–27. 10.1093/biostatistics/kxj037 16632515

[92] Karpievitch Y, Stanley J, Taverner T, Huang J, Adkins JN, Ansong C, et al A statistical framework for protein quantitation in bottom-up MS-based proteomics. Bioinformatics. 2009;25(16):2028–34. 10.1093/bioinformatics/btp362 19535538 PMC2723007

[93] Taverner T, Karpievitch YV, Polpitiya AD, Brown JN, Dabney AR, Anderson GA, et al DanteR: an extensible R-based tool for quantitative analysis of -omics data. Bioinformatics. 2012;28(18):2404–6. 10.1093/bioinformatics/bts449 22815360 PMC3436848

[94] Goeminne LJ, Gevaert K, Clement L. Peptide-level Robust Ridge Regression Improves Estimation, Sensitivity, and Specificity in Data-dependent Quantitative Label-free Shotgun Proteomics. Mol Cell Proteomics. 2016;15(2):657–68. 10.1074/mcp.M115.055897 26566788 PMC4739679

[95] Efstathiou G, Antonakis AN, Pavlopoulos GA, Theodosiou T, Divanach P, Trudgian DC, et al ProteoSign: an end-user online differential proteomics statistical analysis platform. Nucleic Acids Res. 2017;45(W1):W300–W6. 10.1093/nar/gkx444 28520987 PMC5793730

[96] Iurato S, Carrillo-Roa T, Arloth J, Czamara D, Diener-Holzl L, Lange J, et al "DNA Methylation signatures in panic disorder". Transl Psychiatry. 2017;7(12):1287 10.1038/s41398-017-0026-1 29249830 PMC5802688

[97] Durinck S, Moreau Y, Kasprzyk A, Davis S, De Moor B, Brazma A, et al BioMart and Bioconductor: a powerful link between biological databases and microarray data analysis. Bioinformatics. 2005;21(16):3439–40. 10.1093/bioinformatics/bti525 16082012

[98] Plaisier SB, Taschereau R, Wong JA, Graeber TG. Rank-rank hypergeometric overlap: identification of statistically significant overlap between gene-expression signatures. Nucleic Acids Res. 2010;38(17):e169 10.1093/nar/gkq636 20660011 PMC2943622

[99] Alexa A, Rahnenfuhrer J, Lengauer T. Improved scoring of functional groups from gene expression data by decorrelating GO graph structure. Bioinformatics. 2006;22(13):1600–7. 10.1093/bioinformatics/btl140 16606683

[100] Krzywinski M, Schein J, Birol I, Connors J, Gascoyne R, Horsman D, et al Circos: an information aesthetic for comparative genomics. Genome Res. 2009;19(9):1639–45. 10.1101/gr.092759.109 19541911 PMC2752132

[101] Belevich I, Joensuu M, Kumar D, Vihinen H, Jokitalo E. Microscopy Image Browser: A Platform for Segmentation and Analysis of Multidimensional Datasets. PLoS Biol. 2016;14(1):e1002340 10.1371/journal.pbio.1002340 26727152 PMC4699692

[102] Hanley JA, Negassa A, Edwardes MD, Forrester JE. Statistical analysis of correlated data using generalized estimating equations: an orientation. Am J Epidemiol. 2003;157(4):364–75. 10.1093/aje/kwf215 12578807

[103] Allen Institute for Brain Science. Allen Mouse Brain Atlas 2004 [cited 25 January 2019] [Internet]. Available from: http://mouse.brain-map.org/.

[104] Lein ES, Hawrylycz MJ, Ao N, Ayres M, Bensinger A, Bernard A, et al Genome-wide atlas of gene expression in the adult mouse brain. Nature. 2007;445(7124):168–76. 10.1038/nature05453 17151600

