# Supplementary figures and images for: Multi-omics analysis identifies mitochondrial pathways associated with anxiety-related behavior
Source: PLoS Genet. 2019 Sep 26;15(9):e1008358. doi: 10.1371/journal.pgen.1008358 (PMC6762065; doi:10.1371/journal.pgen.1008358)

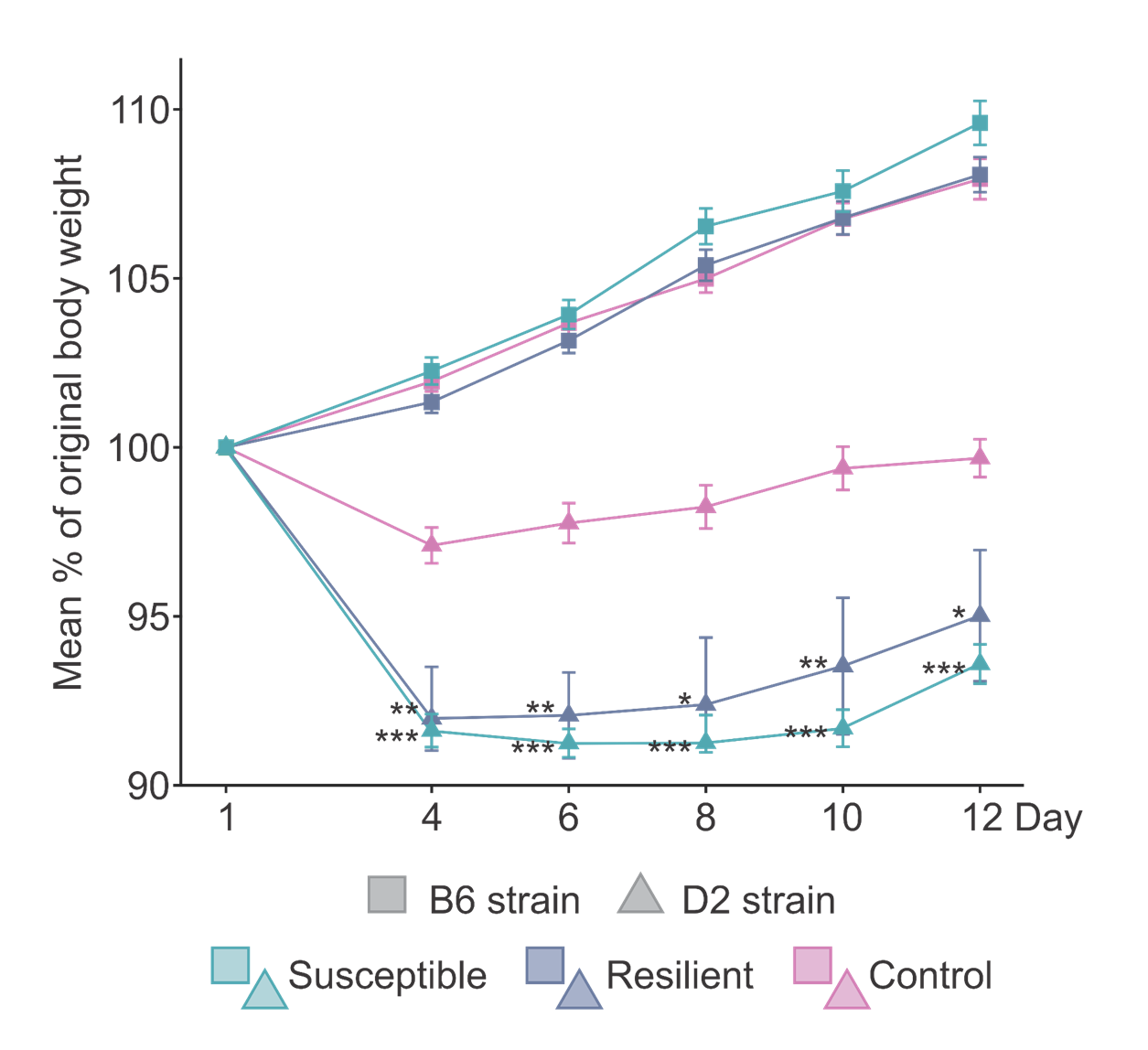

Supplement: S1 Fig — We weighted all mice during CSDS and one day after the SA test. The percentage change in body weight from the baseline (day one) as a function of time is shown (mean ± 1 SEM). In the B6 strain, CSDS did not have a significant effect on body weight as all B6 mice gained weight throughout the duration of the experiment (mixed-design repeated measures ANOVA, F5,157 = 95.34, P = 9.45E-46). Additionally, no differences were observed between the susceptible or resilient mice in comparison to the same-strain controls on any day (one-way ANOVA with Bonferroni post hoc test, Padj > 0.134). Conversely, in the D2 strain, the body weight of all defeated animals decreased during CSDS (mixed-design repeated measures ANOVA, F2,114 = 41.793, P < 0.001). Although all defeated D2 mice weighted less than controls throughout (Bonferroni post hoc test, Padj < 0.010) and up to 48 h after the end of CSDS (Bonferroni post hoc test, Padj < 0.040), the weight of the stress-resilient and stress-susceptible mice did not differ on any day (one-way ANOVA with Bonferroni post hoc test, Padj = 1.000). n = B6: Susceptible = 34, Resilient = 77, Control = 55; D2: Susceptible = 55, Resilient = 6, Control = 59. Outlier criterion: modified Z-score > 3.5. Outliers: n = B6: Resilient = 1, Control = 4; D2: Susceptible = 7, Resilient = 2, Control = 3. *: Padj < 0.05, **: Padj < 0.01, ***: Padj < 0.001. B6: C57BL/6NCrl; D2: DBA/2NCrl. (TIF) [file pgen.1008358.s001.tif]

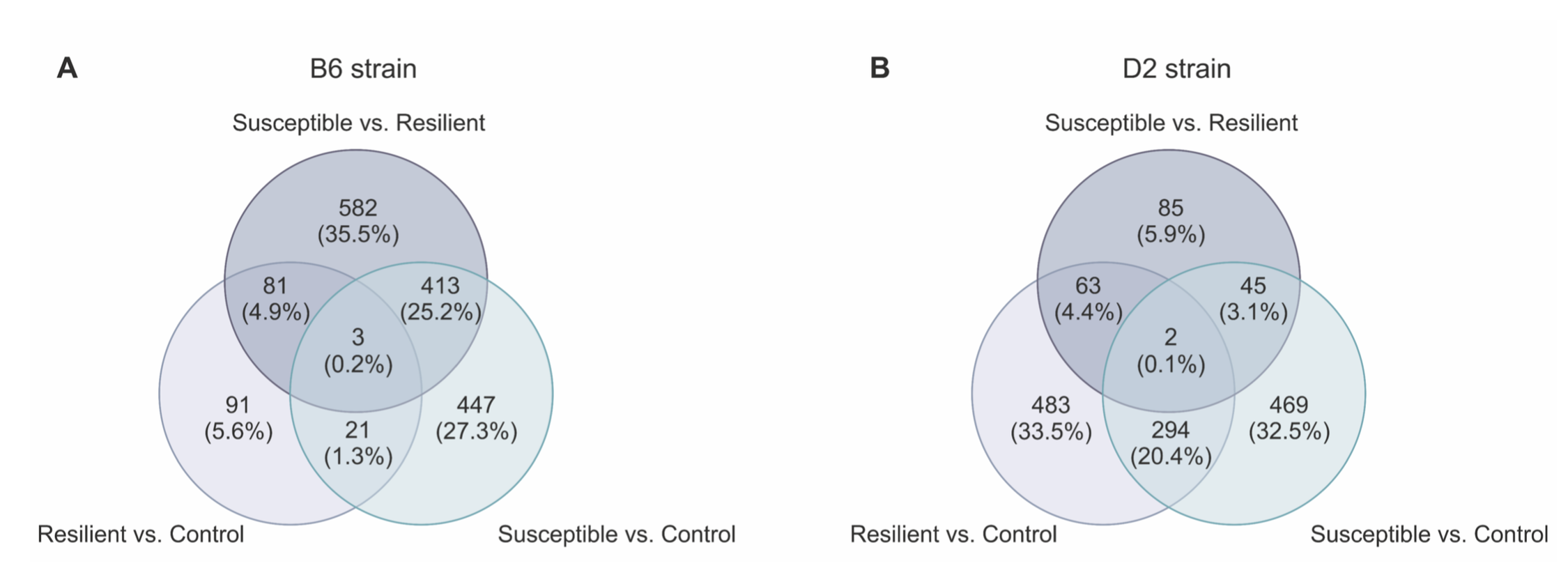

Supplement: S2 Fig — (A-B) Overlap of the differentially expressed (P < 0.05 and |FC| ≥ 1.2) genes between resilient versus control, susceptible versus control and susceptible versus resilient mice, separately in (A) B6 and (B) D2 strains. B6: C57BL/6NCrl; D2: DBA/2NCrl. (TIF) [file pgen.1008358.s002.tif]

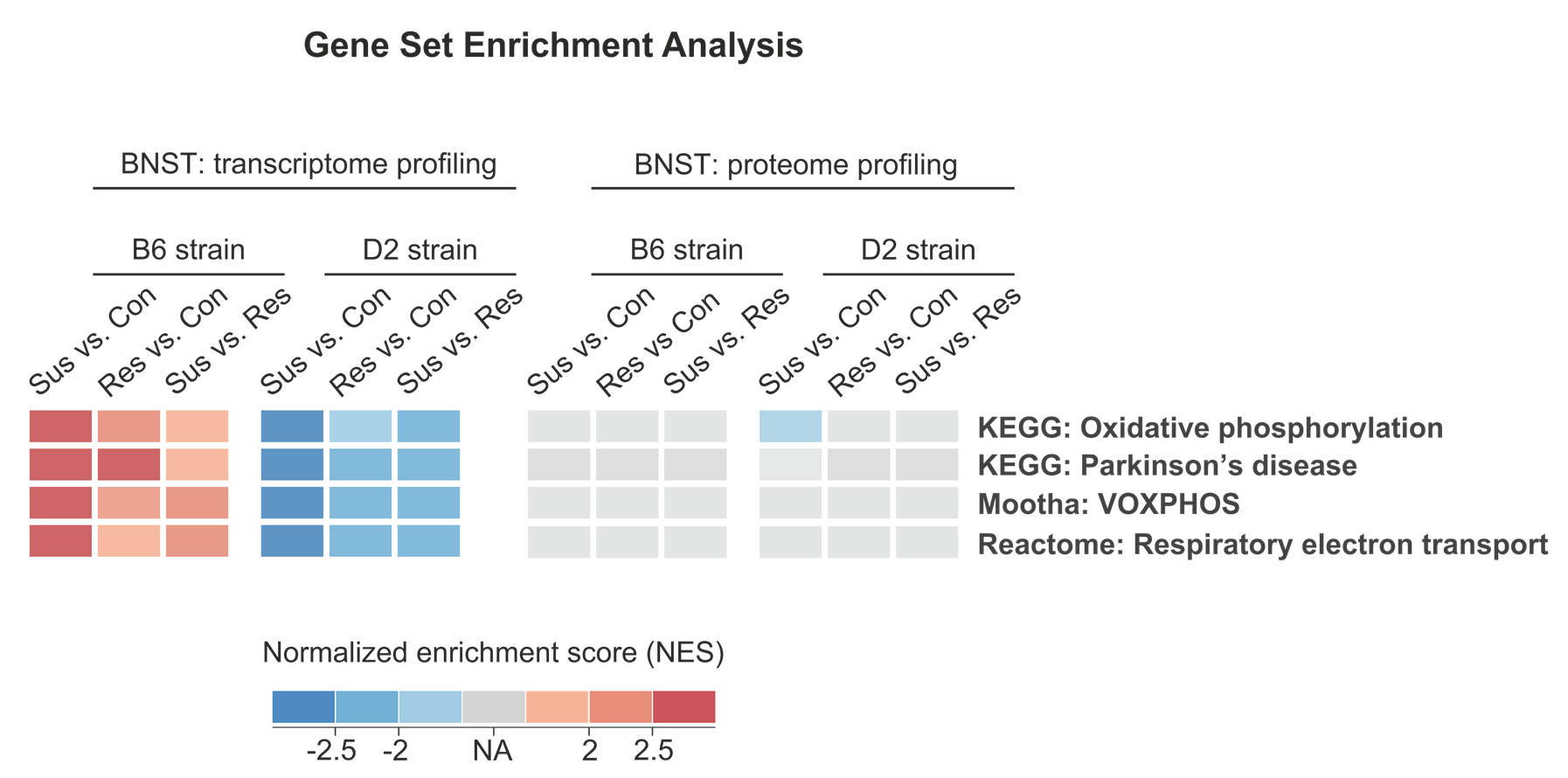

Supplement: S3 Fig — Merged heatmap showing the most significant four overlapping gene sets between the transcriptomic (data set A) and proteomic (data set B) GSEA results in the BNST of CSDS mice. Only significant (PFDR < 0.05) normalized enrichment scores (NES) are shown. Gene sets that did not pass the cut-off are marked in gray (NA). A positive (or negative) NES for a given gene set implicates its overrepresentation at the top (or bottom, respectively) of the ranked list of upregulated (or downregulated, respectively) genes. Gene sets are ordered alphabetically. B6: C57BL/6NCrl; BNST: bed nucleus of the stria terminalis; Con: control; D2: DBA/2NCrl; Res: resilient; Sus: susceptible. (TIF) [file pgen.1008358.s003.tif]

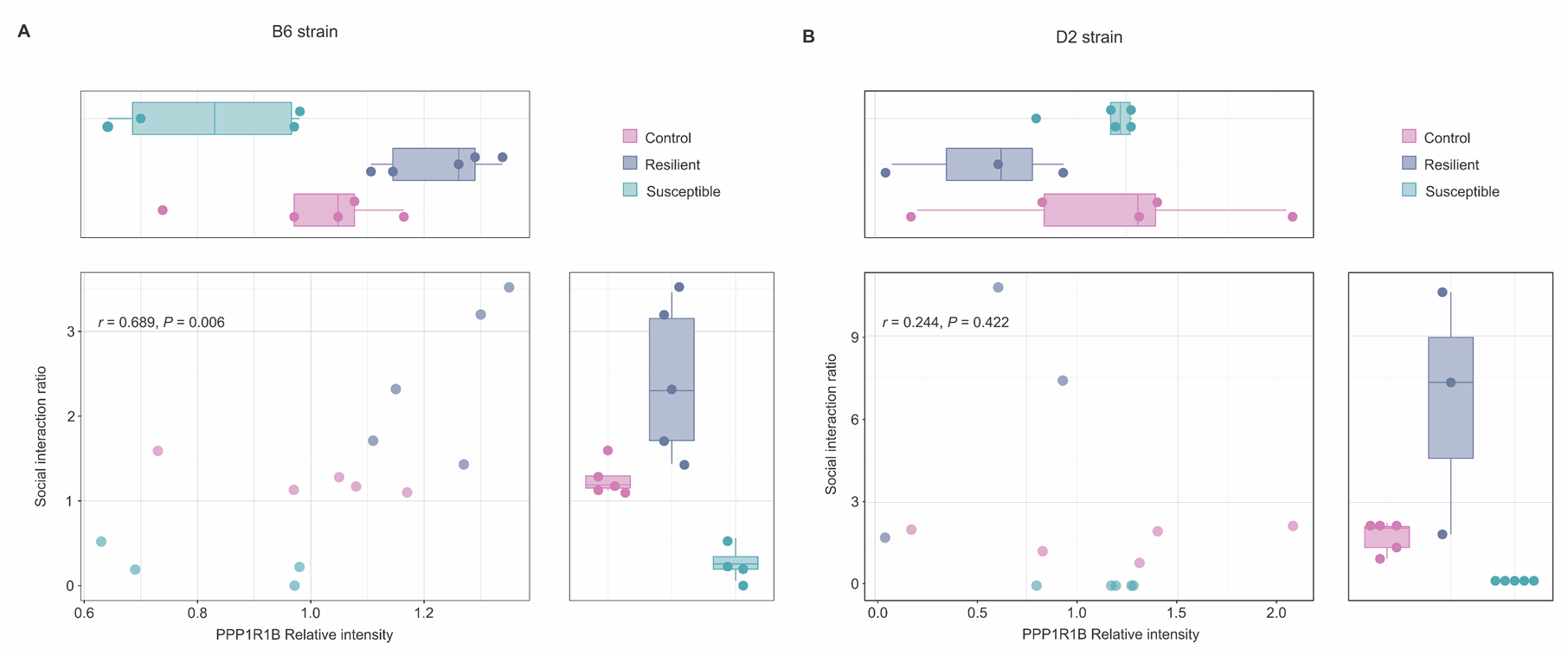

Supplement: S4 Fig — Correlation between PPP1R1B relative intensity in the BNST as detected by Western blot analysis and social interaction (SI) ratios in the (A) B6 strain and (B) D2 strain. B6: C57BL/6NCrl; BNST: bed nucleus of the stria terminalis; D2: DBA/2NCrl; PPP1R1B: protein phosphatase 1 regulatory subunit 1B; r: Pearson correlation coefficient. (TIF) [file pgen.1008358.s004.tif]
